# Astroglial Kir4.1 potassium channel deficit drives neuronal hyperexcitability and behavioral defects in Fragile X syndrome mouse model

**DOI:** 10.1038/s41467-024-47681-y

**Published:** 2024-04-27

**Authors:** Danijela Bataveljic, Helena Pivonkova, Vidian de Concini, Betty Hébert, Pascal Ezan, Sylvain Briault, Alexis-Pierre Bemelmans, Jacques Pichon, Arnaud Menuet, Nathalie Rouach

**Affiliations:** 1 Neuroglial Interactions in Cerebral Physiology and Pathologies, Center for Interdisciplinary Research in Biology, Collège de France, CNRS, INSERM, Labex Memolife, Université PSL, Paris, France; 2 Experimental and Molecular Immunology and Neurogenetics, CNRS UMR7355 and Orléans University, Orléans, France; 3 Department of Genetics, Regional Hospital, Orléans, France; 4 Commissariat à l’Energie Atomique et aux Energies Alternatives (CEA), Département de la Recherche Fondamentale, Institut de biologie François Jacob, MIRCen, and CNRS UMR 9199, Université Paris-Sud, Neurodegenerative Diseases Laboratory, Fontenay-aux-Roses, 92260 France; 5 Present Address: Department of Biomedical Sciences, University of Antwerp, Antwerp, Belgium; 6 Present Address: Department of Physiology, 2nd Faculty of Medicine, Charles University, Prague, Czech Republic

**Keywords:** Astrocyte, Developmental disorders

## Abstract

Fragile X syndrome (FXS) is an inherited form of intellectual disability caused by the loss of the mRNA-binding fragile X mental retardation protein (FMRP). FXS is characterized by neuronal hyperexcitability and behavioral defects, however the mechanisms underlying these critical dysfunctions remain unclear. Here, using male *Fmr1* knockout mouse model of FXS, we identify abnormal extracellular potassium homeostasis, along with impaired potassium channel Kir4.1 expression and function in astrocytes. Further, we reveal that Kir4.1 mRNA is a binding target of FMRP. Finally, we show that the deficit in astroglial Kir4.1 underlies neuronal hyperexcitability and several behavioral defects in *Fmr1* knockout mice. Viral delivery of Kir4.1 channels specifically to hippocampal astrocytes from *Fmr1* knockout mice indeed rescues normal astrocyte potassium uptake, neuronal excitability, and cognitive and social performance. Our findings uncover an important role for astrocyte dysfunction in the pathophysiology of FXS, and identify Kir4.1 channel as a potential therapeutic target for FXS.

## Abstract (web summary)

Fragile X syndrome is a neurodevelopmental disorder with altered neuronal excitability and behavior. Here, the authors show that dysfunction of astroglial Kir4.1 potassium channels drives neuronal and behavioral impairments in a fragile X mouse model.

## Introduction

Fragile X syndrome (FXS) is the most common genetic neurodevelopmental disorder causing intellectual disability and autism spectrum disorders (ASD), with an estimated frequency of around 1:4000 males and 1:8000 females^1^. Patients with FXS exhibit a range of phenotypes including cognitive impairment, social and communication defects, predisposition to epileptic seizures, hypersensitivity to sensory stimuli and macroorchidism^2,3^. This monogenic disorder, presently incurable, is caused by the silencing of the X-linked *Fmr1* gene encoding the fragile X mental retardation protein (FMRP)^4^. The *Fmr1* knockout (KO) mouse is a well-characterized animal model of FXS that mimics many neuronal and behavioral phenotypes observed in FXS patients^3,5^. FMRP binds to numerous mRNAs in neurons and dynamically regulates their transport and translation, especially at the synapse^6,7^. FMRP thereby contributes to the structure and function of synapses^1^. A subset of FMRP-associated mRNAs encode neuronal channels localized at the synapse such as K^+^, Ca^2+^ and *h* channels^6,8–10^. FMRP also directly interacts with neuronal ion channels such as Na^+^-activated K^+^ channels (Slack), Ca^2+^-activated K^+^ channels (BK) and N-type voltage-gated Ca^2+^ channels (Ca_V_2.2), and can modulate their activity^11–13^. Alterations in both the expression and function of these channels can contribute to neuronal hyperexcitability, a prominent feature of FXS^14–16^, and lead to behavioral abnormalities^17^. Despite the large number of identified neuronal mRNA targets of FMRP, the mechanisms underlying the critical dysfunctions in FXS are still unclear and no effective therapy exists so far, making the search for novel targets of prime importance. Interestingly, non-neuronal cells such as astrocytes, the most abundant brain glial cells, intimately interact with neurons at the synaptic and network levels and actively regulate their activities via various mechanisms^18,19^. Astrocytes thus participate to brain physiology and pathologies^20,21^, including several neurodevelopmental disorders such as FXS^22,23^. Astrocytes indeed also express FMRP^24,25^, and its selective deletion in astrocytes increases the number of immature spines in cortical neurons^26^. Furthermore, astroglial FMRP contributes to FXS-related behavioral defects such as learning disability, reduced social recognition and impaired motor learning^27,28^. However, the underlying mechanisms remain largely unknown.

Here we report impaired extracellular K^+^ homeostasis and astroglial Kir4.1 potassium channel function in *Fmr1* KO mice, and identify Kir4.1 mRNA as a binding target of FMRP. Further, we show that restoration of Kir4.1 function selectively to astrocytes in *Fmr1* KO mice reverses the alterations of both neuronal excitability, and cognitive and social behavior. Our findings uncover an important role for astrocyte dysfunction in the pathophysiology of FXS, and identify astroglial Kir4.1 channel as a potential therapeutic target.

## Results

### Increased neuronal excitability and extracellular potassium levels in FMRP-deficient mice

**Fig. 1:**
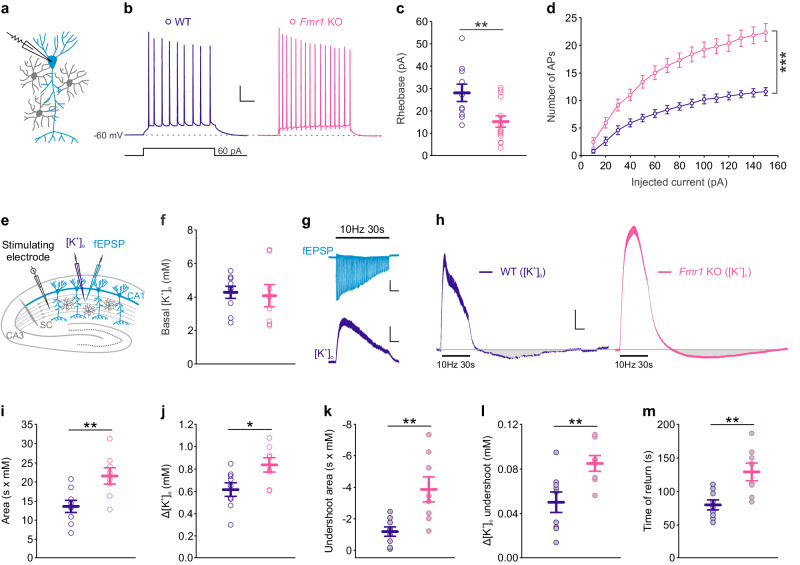
Neuronal hyperexcitability and activity-dependent increase of extracellular potassium levels in *Fmr1* knockout hippocampus. **a** Whole-cell recording from CA1 pyramidal neuron (light blue) surrounded by *stratum radiatum* astrocytes (gray). **b** Representative traces of neuronal responses to 60 pA current pulse in WT (dark blue) and *Fmr1* KO (magenta) in the presence of synaptic blockers (picrotoxin, CPP and NBQX). Scale bar: 20 mV, 100 ms. *Fmr1* KO pyramidal neurons (*n* = 14 neurons from 14 slices in 6 mice) show (**c**), reduced rheobase (*P* = 0.008, t = 2.898, df = 22) and (**d**) increased number of evoked action potentials (APs) as a function of the injected current in comparison to WT neurons (*n* = 10 neurons from 10 slices in 6 mice; *P* < 0.001, F(14, 308) = 18.66). **e** Scheme of simultaneous recordings of extracellular potassium levels ([K^+^]_o_, dark blue) and of field excitatory postsynaptic potentials (fEPSP, light blue) in response to Schaffer collateral (SC) stimulation. **f** [K^+^]_o_ under basal conditions is unchanged in *Fmr1* KO (*n* = 8 slices in 7 mice) as compared to WT hippocampus (*n* = 9 slices in 9 mice; *P* = 0.786, t = 0.276, df = 15). **g** Representative traces of simultaneous recording of fEPSP (light blue) and [K^+^]_o_ (dark blue) in response to 10 Hz, 30 s stimulation of SC. Scale bars, upper panel for fEPSP: 0.2 mV, 5 s; lower panel for [K^+^]_o_: 0.2 mM, 5 s. **h** Representative recordings of [K^+^]_o_ in response to 10 Hz, 30 s stimulation (horizontal bar) in WT (dark blue) and *Fmr1* KO (magenta) hippocampal slices. Scale bar for [K^+^]_o_: 0.1 mM, 10 s. Stimulation of SC induces rise in [K^+^]_o_ showing (**i**), increased area (*P* = 0.006, t = −3.236, df = 15) and (**j**) peak amplitude (*P* = 0.017, t = −2.679, df = 15) in *Fmr1* KO (*n* = 8 slices in 7 mice) as compared to WT mice (*n* = 9 slices in 9 mice). [K^+^]_o_ undershoot (gray) has enlarged (**k**), area (*P* = 0.004, t = 3.449, df = 15), (**l**) peak amplitude (*P* = 0.007, t = −3.112, df = 15) as well as the time of return (*P* = 0.003, t = −3.550, df = 15) in *Fmr1* KO (*n* = 8 slices in 7 mice) in comparison to WT mice (*n* = 9 slices in 9 mice). Data are presented as mean values ± SEM (**c**, **d**, **f**, **i**–**m**). *P < 0.05, **P < 0.01, ***P < 0.001. Two-sided unpaired Student’s *t* test (**c**, **f**, **i**–**m**), Two-way ANOVA repeated measures, *post hoc* Fisher LSD (**d**). CPP: (RS)-3-(2-carboxypiperazin-4-yl)-propyl-1-phosphonic acid; NBQX: 2,3-Dioxo-6-nitro-1,2,3,4-tetrahydrobenzo[f]quinoxaline-7-sulfonamide. Source data are provided as a Source Data file.

The loss of FMRP is associated with neuronal alterations, including membrane excitability, in several brain regions^11,16,17^. Given the importance of the hippocampus in the neuropathology of FXS and its well-defined synaptic organization in the CA1 area, we here focused on this brain region and found enhanced excitability of CA1 pyramidal neurons from *Fmr1* KO male mice, specifically a reduced rheobase (WT: 28.1 ± 3.9 pA, *n* = 10 neurons from 10 slices in 6 mice; *Fmr1* KO: 15.2 ± 2.5 pA, *n* = 14 neurons from 14 slices in 6 mice; *P* = 0.008, two-sided unpaired Student’s *t* test; Fig. 1a–c) and an increased number of evoked action potentials (APs) (*P* < 0.001, two-way ANOVA repeated measures, *post hoc* Fisher LSD; Fig. 1a, b, d) compared to wild type (WT) pyramidal cells. However, membrane potential (V_m_) and membrane resistance (R_m_) of CA1 pyramidal cells remained unchanged (Supplementary Fig. 1a, b). Furthermore, we detected spontaneous firing in ∼30% of the recorded pyramidal cells from *Fmr1* KO mice, in contrast to WT cells, which were silent (Supplementary Fig. 2a, b). Fine regulation of brain extracellular potassium concentration ([K^+^]_o_) is crucial to maintain neuronal excitability^29,30^. We thus investigated whether extracellular K^+^ homeostasis was impaired in the hippocampus of *Fmr1* KO male mice. To do so, we used K^+^-sensitive microelectrodes and measured basal and activity-dependent changes in [K^+^]_o_ in WT and *Fmr1* KO hippocampal slices, specifically in CA1 area *stratum radiatum*, where pronounced activity-dependent [K^+^]_o_ elevation occurs in response to Schaffer collateral stimulation^31–33^ (Fig. 1e–h). We found similar basal [K^+^]_o_ in WT and *Fmr1* KO slices (Fig. 1f; *P* = 0.786, two sided unpaired Student’s *t* test). However, spontaneous variations in [K^+^]_o_ were observed in 25% of *Fmr1* KO hippocampal slices, which did not occur in WT slices (Supplementary Fig. 3a–c). Moreover, simultaneous recordings of [K^+^]_o_ and field potentials (fEPSP) evoked by Schaffer collateral stimulation at 10 Hz for 30 s revealed activity-dependent [K^+^]_o_ transients (Fig. 1e, g) displaying increased area and peak amplitude in *Fmr1* KO as compared to WT hippocampus (WT: 13.656 ± 1479 s × mM and 0.61 ± 0.06 mM, *n* = 9 slices from 9 mice; *Fmr1* KO: 21.618 ± 2014 s x mM and 0.83 ± 0.06 mM, *n* = 8 slices from 7 mice; *P* = 0.006 and *P* = 0.017, two-sided unpaired Student’s *t* test; Fig. 1h–j). Notably, the [K^+^]_o_ response undershoot was more prominent and prolonged in the *Fmr1* KO hippocampus (Fig. 1h), as indicated by its increased area and peak amplitude (WT: −1175 ± 274 s × mM, 0.050 ± 0.009 mM, *n* = 9 slices from 9 mice; *Fmr1* KO: −3856 ± 768 s × mM, 0.085 ± 0.007 mM, *n* = 8 slices from 7 mice; *P* = 0.004 and *P* = 0.007, two-sided unpaired Student’s *t* test; Fig. 1h, k, l). Furthermore, there was a longer time of return to the baseline in *Fmr1* KO relative to WT hippocampus (WT: 80 ± 7 s, *n* = 9 slices from 9 mice; *Fmr1* KO: 129 ± 13 s, *n* = 8 slices from 7 mice; *P* = 0.003, two-sided unpaired Student’s *t* test; Fig. 1h, m), indicating slower recovery of [K^+^]_o_ following neuronal stimulation. Altogether, in addition to enhanced neuronal excitability, our data reveal activity-dependent [K^+^]_o_ accumulation in the hippocampus of FMRP*-*deficient mice.

### Reduced synaptically-evoked K^+^ uptake through Kir4.1 channels in FMRP-deficient astrocytes

**Fig. 2:**
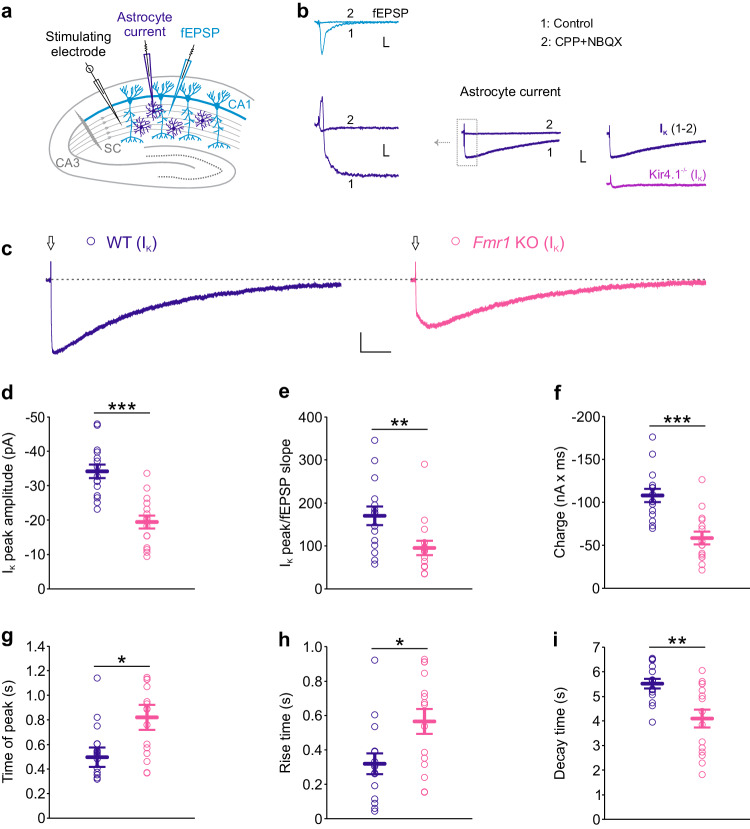
Synaptically-evoked K^+^ uptake through Kir4.1 channels is reduced in *Fmr1* knockout astrocytes. **a** Hippocampus scheme illustrating simultaneous recording of neuronal activity as field excitatory postsynaptic potentials (fEPSPs, light blue) and astrocyte current (dark blue) with extracellular or patch-clamp electrodes, respectively, in response to Schaffer collateral (SC) stimulation. **b** Simultaneous recordings of fEPSP (light blue, trace 1) and synaptically-evoked astrocyte current (dark blue, trace 1) in response to SC stimulation (0.05 Hz) in the presence of picrotoxin (control). Addition of glutamate receptor antagonists CPP and NBQX inhibits fEPSP (light blue, trace 2) and potassium component of astrocyte current (dark blue, trace 2). Subtraction of CPP + NBQX-insensitive component (2) from the total astrocyte current (1) reveals synaptically-evoked astroglial K^+^ current (I_K_). This current is carried by Kir4.1 channels as confirmed by the absence of I_K_ in Kir4.1^−/−^ mice (purple). Scale bars, upper: 0.2 mV, 10 ms; lower left: 10 pA, 10 ms; lower right: 20 pA, 0.2 s. **c** Representative traces of pharmacologically isolated astrocyte I_K_ in WT (dark blue) and *Fmr1* KO mice (magenta). Arrows indicate stimulation artifact. Scale bar: 10 pA, 1 s. Quantification of astrocyte I_K_ properties reveals: (**d**) decrease in I_K_ peak amplitude (*P* < 0.001, t = −5.429, df = 28), (**e**) decrease in I_K_ peak normalized to fEPSP slope (*P* = 0.005, U = 45), (**f**) decrease in charge (*P* < 0.001, U = 24), (**g**) increase in time of peak (*P* = 0.018, t = −2.510, df = 28), (**h**) increase in rise time (*P* = 0.014, t = −2.614, df = 28) and **i** decrease in decay time (*P* = 0.005. U = 44) in *Fmr1* KO (*n* = 15 astrocytes from 15 slices in 11 mice) as compared to WT (*n* = 15 astrocytes from 15 slices in 14 mice). Data are presented as mean values ± SEM (**d**–**i**). *P < 0.05, **P < 0.01, ***P < 0.001. Statistical significance was calculated using two-sided unpaired Student’s *t* test (**d**, **g**, **h**) or two-sided Mann–Whitney test (**e**, **f**, **i**). Source data are provided as a Source Data file.

Synaptic activity is the main source of neuronal K^+^ release, whereas astrocytes are the major players in the clearance of excess K^+^ from the extracellular environment^29,34^. This astroglial function is mediated by the inwardly rectifying potassium channel Kir4.1, which is highly expressed in perisynaptic astroglial processes^35–38^. To examine whether [K^+^]_o_ elevations in *Fmr1* KO hippocampus result from impaired K^+^ uptake via astrocyte Kir4.1 channels, we first performed dual recordings of synaptically-evoked astrocyte current and associated fEPSP in response to single stimulation of Schaffer collaterals at 0.05 Hz (Fig. 2a). Stimulation intensity was set to induce similar fiber volley (FV) amplitudes in hippocampal slices from WT and *Fmr1* KO male mice, which resulted in comparable postsynaptic responses (fEPSPs) (Supplementary Fig. 4a–e). Astrocyte K^+^ current (I_K_) was pharmacologically isolated by addition of glutamate receptor antagonists (CPP and NBQX; Fig. 2b). We confirmed that this current was mediated by astrocyte Kir4.1 channels by the absence of I_K_ in glial conditional Kir4.1^−/−^ mice (Fig. 2b). Quantification of I_K_ properties revealed significant differences between *Fmr1* KO and WT astrocytes. Specifically, astrocyte Kir4.1 currents in *Fmr1* KO astrocytes had reduced peak amplitude, whether normalized or not to fEPSP slope (Normalized data: WT: 169.8 ± 21.6 pA × ms/mV, *n* = 15 astrocytes from 15 slices in 14 mice; *Fmr1* KO: 95.2 ± 16.7 pA × ms/mV, *n* = 15 astrocytes from 15 slices in 11 mice, *P* = 0.005, two-sided Mann–Whitney test; Raw data: WT: −34.2 ± 2.0 pA, *n* = 15, *Fmr1* KO: −19.5 ± 1.9 pA, *n* = 15, *P* < 0.001, two-sided unpaired Student’s *t* test) and reduced charge (WT: −108,100 ± 7683 nA × ms, *n* = 15 astrocytes from 15 slices in 14 mice; *Fmr1* KO: −58,623 ± 7,384 nA × ms, *n* = 15 astrocytes from 15 slices in 11 mice; *P* < 0.001, two-sided Mann–Whitney test) when compared to WT (Fig. 2c–f). Furthermore, we found increased I_K_ time of peak and rise time, whereas decay time was diminished (WT: 554 ± 56 ms, 321 ± 60 ms, 5521 ± 196, *n* = 15 astrocytes from 15 slices in 14 mice; *Fmr1* KO: 785 ± 73 ms, 567 ± 72 ms, 4103 ± 365 ms, *n* = 15 astrocytes from 15 slices in 11 mice; *P* = 0.018 and *P* = 0.014, two-sided unpaired Student’s *t*-test, and *P* = 0.005, two-sided Mann–Whitney test; Fig. 2g–i). These changes in I_K_ in *Fmr1* KO astrocytes were specific, as we found no change in other astrocyte membrane properties, such as current-voltage (I/V) profile, resting membrane potential (RMP) and membrane resistance (R_m_) and gap-junction mediated coupling (Supplementary Fig. 5a–f). Taken together, our data indicate an activity-dependent impairment in Kir4.1-mediated K^+^ clearance from *Fmr1* KO astrocytes.

### Decreased Kir4.1 expression in *Fmr1* KO astrocytes

**Fig. 3:**
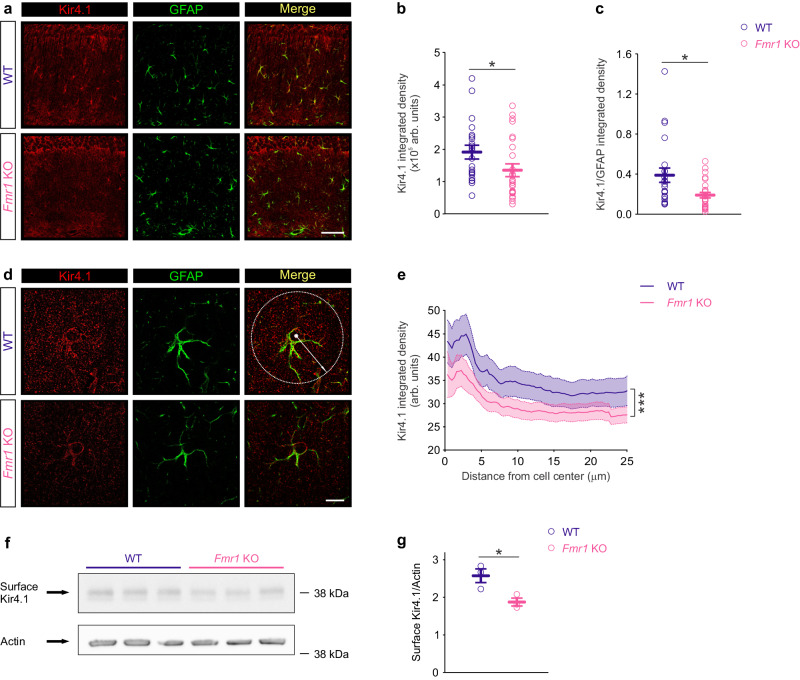
Kir4.1 expression is diminished in *Fmr1* knockout hippocampus. **a** Representative examples of immunofluorescent labeling of Kir4.1 (red) and astrocyte marker glial fibrillary acidic protein (GFAP, green) in WT (dark blue) and *Fmr1* KO (magenta) hippocampus. Scale bar: 50 µm. **b** Quantification of Kir4.1 integrated density (*P* = 0.018, U = 171) and **c** Kir4.1/ GFAP integrated density ratio (*P* = 0.010, U = 161) reveals decreased expression in *Fmr1* KO *stratum radiatum* (*n* = 26 images from 6 mice) as compared to WT (*n* = 22 images from 5 mice). **d** Representative high magnification confocal images of a single astrocyte in CA1 *stratum radiatum* labeled by GFAP (green) and Kir4.1 (red) in WT and *Fmr1* KO mice. Distribution of Kir4.1 puncta was determined at any radial position within 25 µm diameter (white arrow) starting from soma center (white point). Scale bar: 10 µm. **e** Kir4.1 radial intensity profile is similar in *Fmr1* KO (*n* = 29 astrocytes from 5 mice) and WT (*n* = 23 astrocytes from 6 mice) astrocytes, but displays significant shift (*P* < 0.001, F(1, 3892) = 147.1) toward lower Kir4.1 intensity in *Fmr1* KO when compared to WT astrocytes. **f** Examples of western blots showing surface expression of Kir4.1 in WT and *Fmr1* KO hippocampi. Actin was used as a loading control. **g** Decreased level of surface Kir4.1 amount in *Fmr1* KO (*n* = 3 mice; *P* = 0.030, t = 3.314, df = 4) as compared to WT hippocampus (*n* = 3 mice). Data are presented as mean values ± SEM (**b**, **c**, **e**, **g**). *P < 0.05, ***P < 0.001. Statistical significance was assessed by performing two-sided Mann–Whitney test (**b**, **c**), two-way ANOVA, *post hoc* Fisher LSD test (**e**) or two-sided unpaired Student’s *t* test (**g**). Arb. units: arbitrary units. Source data are provided as a Source Data file.

The impairment in Kir4.1 channel function prompted us to examine whether FMRP deficiency alters the expression or distribution of Kir4.1 in astrocytes. We performed immunohistochemistry of Kir4.1 and glial fibrillary acid protein (GFAP) to label astrocytes in hippocampal sections, and found decreased Kir4.1 labeling, normalized or not to GFAP labeling, in CA1 *stratum radiatum* astrocytes from *Fmr1* KO male mice (Kir4.1 integrated density fluorescence: WT: 190.985 ± 19.769 arb. units, *n* = 22 from 5 mice; *Fmr1* KO: 134.871 ± 18.212 arb. units, *n* = 26 from 6 mice, *P* = 0.018, two-sided Mann–Whitney test; Fig. 3a, b; Kir4.1/GFAP integrated density fluorescence ratio: WT: 0.39 ± 0.07 arb. units, *n* = 22 from 5 mice; *Fmr1* KO: 0.19 ± 0.03 arb. units, *n* = 26 from 6 mice, *P* = 0.010, two-sided Mann–Whitney test; Fig. 3c). This decrease in Kir4.1 expression did not result from alteration in astrocyte morphology, since it was unchanged in *Fmr1* KO hippocampus, as demonstrated by morphological reconstructions of whole astrocytes loaded with Alexa-488 (Supplementary Fig. 6a–c). Furthermore, we found no difference in Kir4.1 radial distribution between WT and *Fmr1* KO mice (Fig. 3d, e), as assessed by distribution of Kir4.1 puncta starting from the cell body center along the astroglial processes (Fig. 3d). However, we observed a shift of the distribution curve toward lower values in *Fmr1* KO astrocytes confirming reduced Kir4.1 intensity (*P* < 0.001, two-way ANOVA, *post hoc* Fisher LSD, Fig. 3d, e). This decrease in Kir4.1 expression in *Fmr1* KO mice was region-specific, as it was also found in the somatosensory cortex (Supplementary Fig. 7), but not in the hypothalamus (Supplementary Fig. 8). We next investigated the expression of putative functional Kir4.1 channels in *Fmr1* KO mice. To do so, we performed western blot analysis of membrane Kir4.1 proteins labeled by surface biotinylation, and found decreased Kir4.1 membrane expression in the hippocampus of *Fmr1* KO (WT: 2.57 ± 0.18, *n* = 3 mice; *Fmr1* KO: 1.87 ± 0.11, *n* = 3 mice; *P* = 0.030, two-sided unpaired Student’s t-test; Fig. 3f, g). Altogether, our data demonstrate decreased Kir4.1 expression in *Fmr1* KO hippocampus.

### Astroglial Kir4.1 mRNA is a target of FMRP

**Fig. 4:**
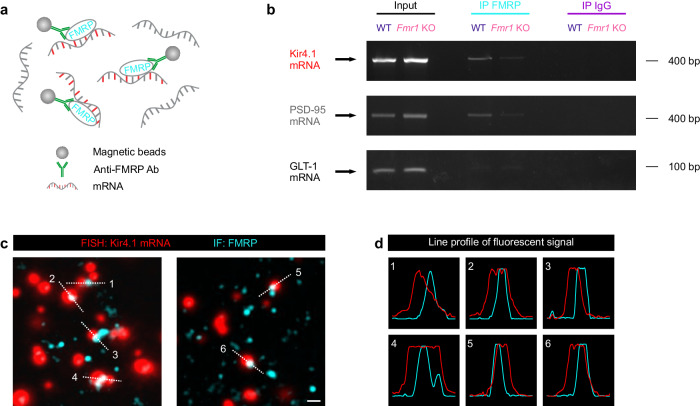
FMRP interacts with Kir4.1 mRNA. **a** Schematic illustration of fragile X mental retardation protein (FMRP, cyan) immunoprecipitation from hippocampus lysates using specific anti-FMRP antibody (green) coupled to magnetic beads (gray). Only mRNAs bound to FMRP are recognized by the antibody-bead complex and are further isolated for RT-PCR analysis. Mouse IgG was used as a negative control. **b** Upper panel: Kir4.1 mRNAs (red) were detected in the FMRP immunoprecipitate (IP, cyan) from WT (dark blue), but not from *Fmr1* KO (magenta) hippocampus. The bands corresponding to Kir4.1 mRNA were identified in the Input (black), and were absent from the immunoglobulin (IgG) immunoprecipitate (purple) of both WT and *Fmr1* KO hippocampus. Middle panel: previously identified FMRP-associated mRNA encoding postsynaptic density protein 95 (PSD-95, gray) was co-immunoprecipitated with FMRP in WT, but not in *Fmr1* KO mice. PSD-95 mRNA was detected in the Input fractions, but was absent from IgG immunoprecipitation complex. Lower panel: glutamate transporter 1 (GLT-1) mRNA was identified in Input, but was not observed in FMRP and IgG immunoprecipitates from both WT and *Fmr1* KO hippocampus. **c** Confocal images of fluorescent in situ hybridization (FISH)-detected Kir4.1 mRNA (red) and immunofluorescence (IF) labeling of FMRP (cyan) in WT hippocampal sections showing their colocalization. Scale bar: 1 µm. **d** Line profiles of individual fluorescent signals along white dashed lines in (**c**) depict colocalization. Experiments were performed in WT mice (*n* = 4) and *Fmr1* KO mice (*n* = 2) (**b**, **c**). Source data are provided as a Source Data file.

The reduction in Kir4.1 expression in astrocytes from *Fmr1* KO male mice and the associated impairment in Kir4.1-mediated K^+^ clearance prompted us to test whether Kir4.1 mRNA is a target of FMRP. FMRP is a RNA-binding protein involved in translation, localization, and stability of various mRNAs^6^. To test whether Kir4.1 mRNA is associated with FMRP, we used a specific anti-FMRP antibody coupled to magnetic beads to immunoprecipitate the FMRP complex from the hippocampus (Fig. 4a), while IgG was used as a negative control. Kir4.1 mRNA was detected in the FMRP immunoprecipitate from WT male mice (Fig. 4b), but not from the *Fmr1* KO mice, as expected. Previously described FMRP-interacting mRNA encoding PSD-95^39^ was used as a positive control, whereas GLT-1 mRNA served as a negative control^24^ (Fig. 4b). Furthermore, to confirm the interaction of FMRP with Kir4.1 mRNA, we combined fluorescent in situ hybridization (FISH) labeling of Kir4.1 mRNA with immunofluorescence of FMRP. This revealed co-localization of fluorescent Kir4.1 mRNA and FMRP in WT hippocampal tissue (Fig. 4c, d). Altogether, these data indicate that astroglial Kir4.1 mRNA is a target of FMRP for translational regulation.

### Viral delivery of Kir4.1 rescues activity-dependent K^+^ uptake in FMRP-deficient astrocytes

**Fig. 5:**
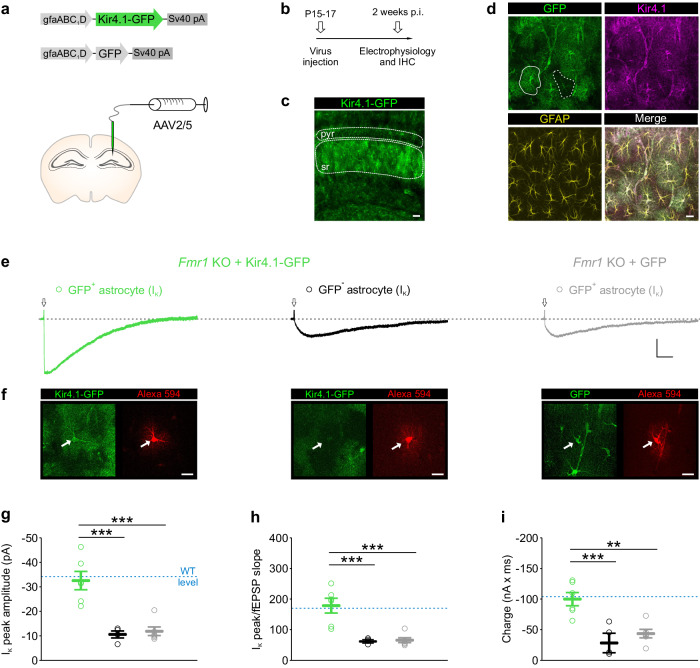
Viral delivery of Kir4.1-GFP into FRMP-deficient astrocytes fully rescues K^+^ uptake. **a** Diagram of adeno-associated vectors (AAV) designed to express Kir4.1 tagged with green fluorescent protein (GFP) or GFP control under the glial fibrillary acidic protein (GFAP) gene promoter (gfaABC_1_D), and scheme of unilateral AAV2/5 microinjection into the mouse hippocampus. **b** Mice were injected at P15-17 and electrophysiology and immunohistochemistry (IHC) were performed 2 weeks post-injection (p.i.). **c** Prominent expression of Kir4.1-GFP transgene (green) in CA1 *stratum radiatum* (sr) after Kir4.1-GFP virus delivery into *Fmr1* KO astrocytes; pyr: pyramidal layer. Experiments were performed in triplicate. Scale bar: 50 µm. **d** Confocal images representing co-immunostaining of GFP (green), Kir4.1 (magenta) and GFAP (yellow) following Kir4.1-GFP virus delivery; white solid line outlines GFP-positive (GFP^+^) *stratum radiatum* astrocytes, whereas white dashed line marks GFP-negative (GFP^−^) astrocyte. Experiments were performed in 5 mice. Scale bar: 20 µm. **e** Representative traces of synaptically-evoked astroglial potassium currents (I_K_) after injection of Kir4.1-GFP (GFP^+^ astrocyte: green; GFP^−^ astrocyte: black) or GFP (GFP^+^ astrocyte: gray) into *Fmr1* KO hippocampus. Stimulation artifacts are indicated by arrows. Scale bar: 10 pA, 1 s. **f** GFP^+^ and GFP^−^ astrocytes were loaded with Alexa 594 dye (red) during I_K_ recording. Scale bar: 20 µm. Delivery of Kir4.1-GFP into *Fmr1* KO astrocytes significantly restores (**g**) I_K_ peak amplitude (*P* < 0.0001, F(2, 13) = 20.50), (**h**) I_K_ peak amplitude normalized to field excitatory postsynaptic potential (fEPSP) slope (*P* = 0.0003, F(2, 13) = 16.12) and (**i**) I_K_ charge (*P* = 0.0008, F(2, 13) = 13.11) to the level observed in WT astrocytes (blue dashed line). Number of recorded and Alexa 594 loaded cells (**f**–**i**): Kir4.1-GFP-injected GFP^+^ astrocytes, *n* = 6 astrocytes from 6 slices in 5 mice (green); Kir4.1-GFP-injected GFP^-^ astrocytes, *n* = 4 astrocytes from 4 slices in 3 mice (black); GFP-injected GFP^+^ astrocytes, *n* = 6 astrocytes from 6 slices in 4 mice (gray). Data are presented as mean values ± SEM (**g**–**i**). **P < 0.01, ***P < 0.001. Statistical significance was calculated using one-way ANOVA, *post hoc* Fisher LSD test (**g**–**i**). Source data are provided as a Source Data file.

We tested whether viral delivery of Kir4.1 channels specifically to hippocampal astrocytes can rescue normal astrocyte K^+^ uptake in *Fmr1* KO mice. To this end, we delivered adeno-associated virus serotype 2/5 (AAV2/5) to express either GFP-tagged Kir4.1 (AAV2/5 Kir4.1-GFP) or GFP (AAV2/5 GFP) only, as a control, under the GFAP promoter (gfaABC1D), in the hippocampus of *Fmr1* KO male mice at P15-17 (Fig. 5a, b). Two weeks after viral injection, prominent expression of GFP was observed in the hippocampal CA1 region (Fig. 5b, c). The specificity of viral transduction in astrocytes was verified by co-immunolabeling of GFP, GFAP and Kir4.1 (Fig. 5d). Single cell analysis in *Fmr1* KO mice transduced with AAV2/5 Kir4.1-GFP in hippocampal astrocytes revealed that Kir4.1 expression in GFP-positive (GFP^+^) astrocytes was significantly increased by ∼50%, as compared to injection of AAV2/5 GFP (Supplementary Fig. 9a, b). Almost all GFP^+^ cells expressed GFAP, confirming specific viral targeting of astrocytes (Supplementary Fig. 9a, c). In addition, we found that ∼70% of GFAP^+^ astrocytes in CA1 *stratum radiatum* were targeted for viral transduction and expressed GFP (Supplementary Fig. 9a, d). We then assessed the functional properties of synaptically-evoked astrocyte I_K_ after viral transduction of Kir4.1-GFP (Fig. 5e). Alexa 594 dye was systematically loaded into astrocytes via the patch pipette to further identify the patched cell as either GFP^+^ or GFP-negative (GFP^-^) (Fig. 5f). We found that viral delivery of Kir4.1-GFP in astrocytes from *Fmr1* KO mice restored to WT levels the peak amplitude, normalized or not to fEPSP slope, and charge of synaptically-evoked I_K_ in GFP^+^ astrocytes, whereas GFP^-^ astrocytes from the same animal retained reduced I_K_, as detected in uninjected *Fmr1* KO mice (Kir4.1-GFP-injected, GFP^+^ astrocytes: −32.5 ± 3.7 pA, 178.5 ± 24.3 pA × ms/mV, −99.918 ± 10,795 nA × ms, *n* = 6 astrocytes from 6 slices in 5 mice; Kir4.1-GFP-injected, GFP^-^ astrocytes: −10.6 ± 1.4 pA, 62.4 ± 3.6 pA × ms/mV, −28.240 ± 15.917 nA × ms, *n* = 4 astrocytes from 4 slices in 3 mice; *P* < 0.001, one-way ANOVA, *post hoc* Fisher LSD test; Fig. 5e–i and Fig. 2c–f). Furthermore, delivery of the GFP control virus into *Fmr1* KO astrocytes had no effect on synaptically-evoked I_K_, which remained similar to I_K_ in uninjected *Fmr1* KO mice (GFP-injected: −11.9 ± 1.8 pA, 65.6 ± 7.9 pA × ms/mV, −43.554 ± 6.966 nA × ms, *n* = 6 astrocytes from 6 slices in 4 mice; *P* < 0.001, one-way ANOVA, *post hoc* Fisher LSD test; Fig. 5e–i and Fig. 2c–f). Taken together, our data demonstrate complete rescue of activity-dependent astrocyte K^+^ uptake after viral delivery of Kir4.1 in *Fmr1* KO hippocampal astrocytes.

### Viral delivery of Kir4.1 in astrocytes normalizes neuronal excitability in *Fmr1* knockout mice

**Fig. 6:**
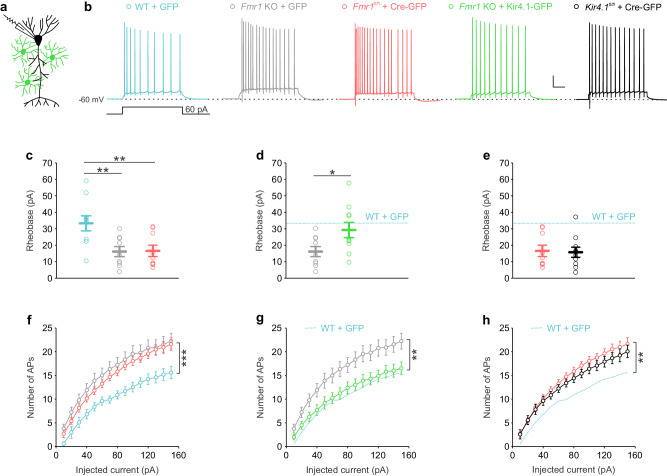
Neuronal excitability is rescued by viral delivery of Kir4.1-GFP in astrocytes of *Fmr1* knockout mice. **a** Whole-cell recording from CA1 pyramidal neuron (black) surrounded by green fluorescent protein (GFP)-positive astrocytes (green). **b** Representative traces of neuronal voltage responses evoked by 60 pA current pulse in WT mice injected with GFP (WT + GFP, light blue), *Fmr1* KO mice injected with GFP (*Fmr1* KO + GFP, gray), *Fmr1*^fl/fl^ mice injected with Cre-GFP (*Fmr1*^fl/fl^ + Cre-GFP, red), *Fmr1* KO mice injected with Kir4.1-GFP (*Fmr1* KO + Kir4.1-GFP, green) and *Kir4.1*^*fl/fl*^ mice injected with Cre-GFP (*Kir4.1*^*fl/fl*^ + Cre-GFP, black). Scale bar: 20 mV, 100 ms. **c** Rheobases of pyramidal neurons in *Fmr1* KO + GFP and *Fmr1*^fl/fl^ + Cre-GFP mice are significantly decreased in comparison to WT + GFP mice (light blue dashed line, *P* = 0.005, F(2, 25) = 6.739). **d** Rheobase of neurons in *Fmr1* KO + Kir4.1-GFP mice is increased in comparison to *Fmr1* KO + GFP mice and reached the values found in WT + GFP mice (*P* = 0.024, F(2, 26) = 4.343). **e** Neurons from *Fmr1*^fl/fl^ + Cre-GFP and *Kir4.1*^*fl/fl*^ + Cre-GFP mice display similar rheobases, which are decreased in comparison to WT + GFP mice (*P* = 0.004, F(2, 28) = 6.648). **f** The number of evoked action potentials (APs) is significantly higher in neurons from *Fmr1* KO + GFP and *Fmr1*^fl/fl^+Cre-GFP mice in comparison to WT + GFP (*P* = 0.0008, F(2, 25) = 9.715). **g** Delivery of Kir4.1-GFP into *Fmr1* KO astrocytes fully restores the number of evoked APs to the level observed in WT + GFP mice (*P* = 0.0023, F(2, 26) = 7.754). **h** Neurons from *Fmr1*^fl/fl^ + Cre-GFP and *Kir4.1*^*fl/fl*^ + Cre-GFP mice display similar number of evoked APs, which are increased in comparison to neurons from WT + GFP (*P* = 0.0014, F(2, 28) = 8.434). Number of recorded cells (**c**–**h**): WT + GFP (*n* = 10 neurons from 10 slices in 4 mice), *Fmr1* KO + GFP (*n* = 9 neurons from 9 slices in 6 mice), *Fmr1*^fl/fl^ + Cre-GFP (*n* = 9 neurons from 8 slices in 4 mice), *Fmr1* KO + Kir4.1-GFP (*n* = 10 neurons from 9 slices in 6 mice), *Kir4.1*^*fl/fl*^ + Cre-GFP (*n* = 12 neurons from 10 slices in 7 mice). Data are presented as mean values ± SEM (**c**–**h**). *P < 0.05, **P < 0.01. Statistical significance was assessed by performing one-way ANOVA, *post hoc* Fisher LSD test (**c**–**e**) and repeated measures two-way ANOVA, *post hoc* Fisher LSD test (**f**–**h**). Source data are provided as a Source Data file.

We then tested whether the neuronal hyperexcitability in FXS results from astroglial FMRP deficiency and impairment in Kir4.1-mediated K^+^ uptake. To do so, we first conditionally knocked out FMRP specifically in astrocytes, using viral delivery of Cre-GFP under the GFAP promoter into hippocampal astrocytes from *Fmr1*^fl/fl^ male mice (P15-17), and recorded two weeks later CA1 pyramidal neurons surrounded by GFP^+^ astrocytes using whole-cell patch-clamp (Fig. 6a). Conditional knockout of astroglial FMRP was sufficient to significantly reduce the rheobase and increase the number of evoked APs of CA1 pyramidal neurons to the level observed in GFP-transduced *Fmr1* KO male mice (Fig. 6a–d). To test the implication of impaired Kir4.1-mediated K^+^ uptake in astrocytes, we then restored Kir4.1 expression specifically in hippocampal astrocytes deficient for FMRP, using viral delivery of Kir4.1-GFP under the GFAP promoter in the hippocampus of *Fmr1* KO mice (Supplementary Fig. 9). Remarkably, expression of the Kir4.1-GFP transgene in *Fmr1* KO astrocytes fully restored to WT levels (GFP-injected) the rheobase and number of evoked APs in CA1 pyramidal neurons (rheobase: WT + GFP: 33.3 ± 4.6 pA, *n* = 10 neurons from 10 slices in 4 mice; *Fmr1* KO + GFP: 16.2 ± 3.1 pA, *n* = 9 neurons from 9 slices in 6 mice; *Fmr1*^fl/fl^ + Cre-GFP: 16.7 ± 3.4 pA, *n* = 9 neurons from 8 slices in 4 mice; *Fmr1* KO + Kir4.1-GFP: 29.3 ± 4.6 pA, *n* = 10 neurons from 9 slices in 6 mice; *P* = 0.024, one-way ANOVA, *post hoc* Fisher LSD test; evoked APs: *P* = 0.002, two-way ANOVA repeated measures, *post hoc* Fisher LSD test; Fig. 6a–d). Membrane potential and membrane resistance remained similar in all the tested experimental conditions (Supplementary Fig. 10), and the GFP control virus (AAV2/5-GFP) injected into WT and *Fmr1* KO hippocampi had no effect by itself on neuronal excitability (Fig. 6b–d and Fig. 1b–d). To confirm the involvement of astrocyte Kir4.1 deficit in neuronal hyperexcitability in FXS, we induced Kir4.1 deletion specifically in hippocampal astrocytes by viral delivery of Cre-GFP under the GFAP promoter into the hippocampus of *Kir4.1*^*fl/fl*^ male mice (P15–17; Supplementary Fig. 11). Two weeks after the virus injection, we performed co-immunolabeling of GFP, GFAP and Kir4.1, which showed pronounced expression of GFP together with the absence of Kir4.1 expression (Supplementary Fig. 11). We found that the inducible conditional deletion of Kir4.1 specifically in astrocytes mimicked the hyperexcitability of CA1 pyramidal cells observed in mice with conditional astroglial deletion of FMRP *(Fmr1*^fl/fl^ mice injected with Cre-GFP under the GFAP promoter), as shown by the similar decreased rheobase and increased number of action potentials in both conditions compared to GFP-injected WT mice (rheobase: *Kir4.1*^*fl/fl*^ + Cre-GFP: 17.2 ± 2.9 pA, *n* = 12 neurons from 10 slices in 7 mice; *Fmr1*^fl/fl^ + cre-GFP: 16.7 ± 3.4 pA, *n* = 9 neurons from 8 slices in 4 mice; WT + GFP: 33.3 ± 4.6 pA, *n* = 10 neurons from 10 slices in 4 mice; *P* = 0.004, one-way ANOVA, *post hoc* Fisher LSD test; number of APs: *P* = 0.0014, two-way ANOVA repeated measures, *post hoc* Fisher LSD; Fig. 6e, h). Altogether, our data indicate that FMRP deficiency in astrocytes induces neuronal hyperexcitability that can be rescued by restoring Kir4.1 channels in astrocytes from FMRP-deficient mice.

### Restoring astrocyte Kir4.1 rescues behavioral deficits in *Fmr1* knockout mice

**Fig. 7:**
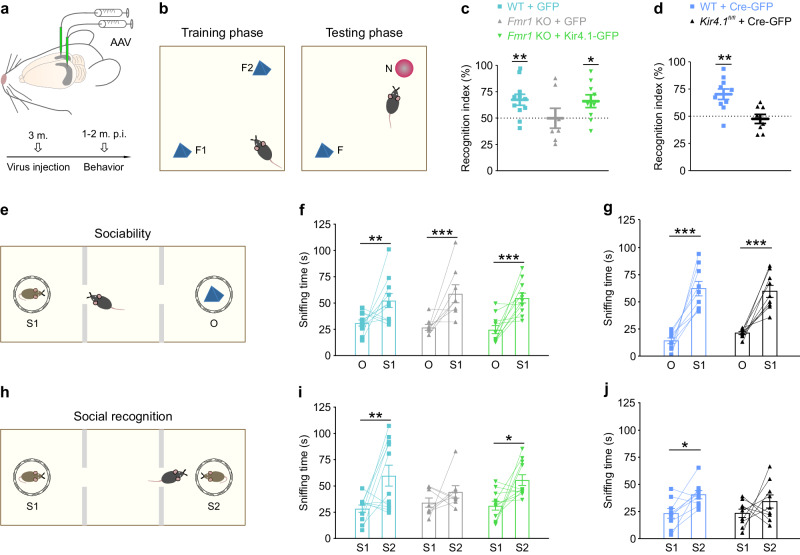
Viral delivery of Kir4.1-GFP in *Fmr1* knockout astrocytes rescues hippocampus-dependent cognitive and social defects. **a** Adeno associated virus (AAV) bilateral injection into the hippocampus of 3-month-old mice. Behavioral testing was performed 1−2 months post injection (p.i.). **b** Novel object recognition test composed of training phase when mice were exposed to two identical objects (F1 and F2) and testing phase whereas one familiar object (F) was replaced by a novel object (N). **c** In the testing phase, WT mice injected with green fluorescent protein (GFP; WT + GFP, *n* = 11 mice; light blue) exhibited a preference for the N (*P* = 0.007, t = 3.356, df=10), while GFP-injected *Fmr1* KO mice (*Fmr1* KO + GFP, *n* = 7 mice; gray) had a similar preference (*P* = 0.991, t = 0.01219, df = 6) for the F and the N. Delivery of Kir4.1-GFP into *Fmr1* KO (*Fmr1* KO + Kir4.1-GFP, *n* = 9 mice; green) hippocampal astrocytes restored (*P* = 0.030, t = 2.636, df = 8) the preference for the N. **d** Cre-GFP-injected WT mice (WT + Cre-GFP, *n* = 10 mice; blue) show higher recognition index (*P* = 0.002, t = 4.193, df = 9) for the N, whereas *Kir4.1*^*fl/fl*^ mice injected with Cre-GFP (*Kir4.1*^*fl/fl *^+ Cre-GFP, *n* = 8 mice; black) do not display preference for N (*P* = 0.565, t = 0.603, df = 7). **e**, **h** Test mouse was introduced to stranger mouse (S1) and object (O) during the sociability phase and then the object was replaced by a novel stranger mouse (S2) during the social recognition phase. **f**, **g** Mice in each experimental group spend more time sniffing the S1 than the O (**f**: WT + GFP, *n* = 11 mice; *Fmr1* KO + GFP, *n* = 8 mice; *Fmr1* KO + Kir4.1-GFP, *n* = 11 mice; *P* < 0.001, F(1, 54) = 43.57; **g**: WT + Cre-GFP, *n* = 9 mice; *Kir4.1*^*fl/fl*^ + Cre-GFP mice, *n* = 9 mice; *P* < 0.001, F(1, 32) = 89.13). (**i**) WT + GFP mice (*n* = 11 mice) displayed preference for S2 mouse whereas *Fmr1* KO + GFP mice (*n* = 8 mice) failed to distinguish the S1 and S2 mice. This social interaction impairment was corrected after Kir4.1-GFP injection into the hippocampus of *Fmr1* KO mice (*n* = 11 mice; *P* < 0.001, F(1, 54) = 19.02). WT + Cre-GFP mice (*n* = 9 mice) show preference for S2, whereas *Kir4.1*^*fl/fl*^ + Cre-GFP mice (*n* = 9 mice) failed to distinguish the S1 and S2 mice (*P* = 0.005, F(1, 32) = 9.093). Data are presented as mean values ± SEM (**c**, **d**, **f**, **g**, **i**, **j**). *P < 0.05, **P < 0.01, ***P < 0.001. Two-sided one sample *t*-tests to 50% (**c**, **d**) and Two-way ANOVA followed by a Sidak *post hoc* test (**f**, **g**, **i**, **j**). Source data are provided as a Source Data file.

Individuals with FXS display a number of behavioral deficits including hippocampus-dependent cognitive and social impairments^1,3^, which are recapitulated in the *Fmr1* KO mouse model^3^. To test whether bilateral delivery of Kir4.1-GFP into hippocampal astrocytes (Fig. 7a) can rescue cognitive impairment in *Fmr1* KO male mice, we employed the novel object recognition (NOR) test. NOR test is used to measure recognition memory based on the mouse natural preference for novelty^40^. Male mice were introduced to two identical objects in the training phase, and thereafter one familiar object was replaced by a novel object in the testing phase (Fig. 7b). Recognition memory was then assessed by the relative time spent exploring a novel versus familiar object. During the training phase, *Fmr1* KO mice injected with Kir4.1-GFP displayed similar recognition index for the two identical objects compared to WT and *Fmr1* KO mice injected with GFP (Supplementary Fig. 12). However, in the testing phase, WT mice injected with GFP displayed preference for the novel object, whereas *Fmr1* KO mice injected with GFP failed to distinguish between the familiar and the novel object (WT + GFP, 67.5 ± 5.2%, *n* = 11 mice; *Fmr1* KO + GFP, 49.9 ± 9.5%, *n* = 7 mice; two sided one sample *t*-test vs. 50%: WT + GFP: *P* = 0.007; *Fmr1* KO + GFP: *P* = 0.991; Fig. 7c). These data are in line with previous findings showing reduced preference for novelty in *Fmr1* KO mice^41,42^. Delivery of Kir4.1-GFP into *Fmr1* KO astrocytes rescued recognition memory impairment to WT levels (GFP-injected WT mice) (*Fmr1* KO + Kir4.1-GFP, 66.2 ± 6.1%, *n* = 9 mice; two-sided one sample *t*-test vs. 50%: *Fmr1* KO + Kir4.1-GFP, *P* = 0.030; Fig. 7c), indicating that astroglial Kir4.1 channel normalized cognitive performance in *Fmr1* KO mice. To confirm the involvement of astrocyte Kir4.1 deficit in the impairment of recognition memory in FXS, we induced the conditional deletion of Kir4.1 specifically in hippocampal astrocytes by viral delivery of Cre-GFP under the GFAP promoter into the hippocampus of 3 month-old *Kir4.1*^*fl/fl*^ male mice, and tested these mice for NOR. During the training phase, both WT and *Kir4.1*^*fl/fl*^ mice transduced with Cre-GFP in astrocytes showed similar recognition of two identical objects (Supplementary Fig. 12). However, in testing phase, whereas WT mice injected with Cre-GFP displayed preference for novel object, *Kir4.1*^*fl/fl*^ mice injected with Cre-GFP failed to distinguish novel from familiar object (WT + Cre-GFP: 70.0 ± 4.8%, *n* = 10 mice; *Kir4.1*^*fl/fl*^ + Cre-GFP: 47.53 ± 4.2%, *n* = 8 mice; two sided one sample t-tests to 50%, WT + Cre-GFP: *P* = 0.002; *Kir4.1*^*fl/fl*^ + Cre-GFP: *P* = 0.565; Fig. 7d), thus mimicking the impaired cognitive behavior of *Fmr1* KO mice injected with GFP (Fig. 7c).

FXS patients exhibit difficulties in social communication^1,3^ that are found in *Fmr1* KO mice as impaired ability to discriminate social novelty^3,43^. To assess cognitive aspect of mice social interactions, we used the three-chamber test, which is based on the mouse preference for social novelty^3^. During sociability phase, test mice were exposed to stranger mouse and object (Fig. 7e), while during social recognition phase, the object was replaced by a novel stranger mouse (Fig. 7h). WT and *Fmr1* KO male mice injected with GFP, as well as *Fmr1* KO mice injected with Kir4.1-GFP displayed a preference for the stranger mouse over the object (WT + GFP, O: 31.0 ± 2.9, S1: 52.4 ± 6.5, *n* = 11 mice; *Fmr1* KO + GFP, O: 26.8 ± 2.8, S1: 58.8 ± 8.6, *n* = 8 mice; *Fmr1* KO + Kir4.1-GFP, O: 24.6 ± 3.8, S1: 54.7 ± 4.7, *n* = 11 mice; *P* < 0.001, two-way ANOVA, *post hoc* Sidak test; object and stranger 1 mouse: F(1, 54) = 43.57, *P* < 0.001; experimental groups: F(2, 54) = 0.19, *P* = 0.83; interaction: F(2, 54) = 0.62, *P* = 0.54; Fig. 7f). In the social recognition phase, while the GFP-injected WT mice interacted more with the novel (S2) than the familiar stranger (S1) mouse, GFP-injected *Fmr1* KO mice failed to distinguish between familiar and novel stranger mouse (WT + GFP, S1: 28.4 ± 3.5 s, S2: 59.8 ± 10.0 s, *n* = 11 mice; *Fmr1* KO + GFP, S1: 34.1 ± 4.4 s, S2: 44.3 ± 5.9 s, *n* = 8 mice; Fig. 7i). However, when *Fmr1* KO astrocytes were transduced with Kir4.1-GFP, the preference to explore the novel stranger mouse was restored to WT levels (WT mice injected with GFP) (*Fmr1* KO + Kir4.1-GFP, S1: 31.2 ± 4.2 s, S2: 55.6 ± 5.1 s, *n* = 11 mice; *P* < 0.001, two-way ANOVA, *post hoc* Sidak test; stranger mice: F(1, 54) = 19.02; experimental groups: F(2, 54) = 0.33, *P* = 0.72; interaction: F(2, 54) = 1.42, *P* = 0.25; Fig. 7i). It is noteworthy that Kir4.1 deficiency of hippocampal astrocytes from *Kir4.1*^*fl/fl *^ mice transduced in their hippocampi with Cre-GFP under the GFAP promoter, did not alter their preference for the stranger mouse over the object during the sociability phase, as compared to WT mice transduced with Cre-GFP in hippocampal astrocytes (WT + Cre-GFP, O: 14.2 ± 2.7 s, S1: 62.3 ± 6.6 s, *n* = 9 mice; *Kir4.1*^*fl/fl *^+ Cre-GFP, O: 21.3 ± 1.3 s, S1: 59.8 ± 5.6 s, *n* = 9 mice; *P* < 0.001, two-way ANOVA, *post hoc* Sidak test; Fig. 7g). However, in the social recognition phase, *Kir4.1*^*fl/fl*^ mice injected with Cre-GFP failed to distinguish the familiar and novel stranger mouse, thus mimicking the impaired social behavior of *Fmr1* KO mice injected with GFP (Fig. 7i), in contrast to Cre-GFP injected WT mice, which displayed preference for novel stranger mouse (WT + Cre-GFP, S1: 23.2 ± 4.5 s, S2: 40.5 ± 3.8 s, *n* = 9 mice; *Kir4.1*^*fl/fl *^+ Cre-GFP, S1: 23.4 ± 3.9 s, S2: 34.2 ± 6.1 s, *n* = 9 mice; *P* = 0.005, two-way ANOVA, *post hoc* Sidak test; Fig. 7j). Taken together, our data demonstrate that Kir4.1 deficit in *Fmr1* KO mice contributes to hippocampus-dependent cognitive and social abnormalities, which can be corrected by restoring Kir4.1 channels in *stratum radiatum* astrocytes. This points to astroglial Kir4.1 channel as a promising therapeutic target to ameliorate FXS-associated behavioral phenotypes.

## Discussion

Our study indicates that astrocytes play an important role in the pathophysiology of FXS by affecting neuronal output and behavioral phenotype. Along with increased excitability of CA1 pyramidal neurons, we demonstrated activity-dependent [K^+^]_o_ accumulation in the hippocampus of *Fmr1* KO male mice, indicating disturbance of extracellular K^+^ homeostasis in FXS. This is associated with reduced ability of astrocytes to uptake excess K^+^ released during synaptic activity via Kir4.1 channels, whose expression is decreased. Furthermore, we identified Kir4.1 mRNA as a target of FMRP. Finally, viral-mediated expression of Kir4.1 in astrocytes rescued several neuronal and behavioral abnormalities in *Fmr1* KO male mice. Together, our findings establish defective astrocyte Kir4.1 channels as an important contributor to neuronal hyperexcitability and behavioral impairments in FXS.

Astrocytes are essential elements of the tripartite synapse that dynamically regulate the extracellular environment. Specifically, they control elevated [K^+^]_o_ following neuronal activity through K^+^ clearance^29,34^. Here we demonstrate that astrocyte K^+^ uptake through Kir4.1 channels during synaptic activity is markedly reduced in *Fmr1* KO male mice. The magnitude of synaptically-evoked K^+^ current in *Fmr1* KO astrocytes exhibits a reduction of ∼45%. Moreover, we found that I_K_ kinetics displays prolonged rise time and time of peak, whereas decay time was shorter in *Fmr1* KO male mice. Impaired ability of astrocytes to remove excess K^+^ through Kir4.1 in FXS is consistent with previously reported findings in glial conditional Kir4.1^-/-^ mice. Astrocyte K^+^ current evoked by single stimulation of Schaffer collaterals is indeed almost completely abolished in the hippocampus of glial conditional Kir4.1^-/-^ mice^35,37^. Despite the reduction by ∼45% of the Kir4.1 currents in astrocytes from *Fmr1* KO mice, we found that basal [K^+^]_o_ and astroglial RMP remain intact, in contrast to the depolarized RMP reported in astrocytes deficient for Kir4.1^35,44^. This suggests that the remaining Kir4.1 activity, representing ∼55% of the current, is sufficient to maintain basal [K^+^]_o_ and normal astroglial RMP, but however fails to ensure efficient clearance of excess K^+^ released during neuronal activity.

It is important to note that we found activity-dependent [K^+^]_o_ accumulation and in particular enlarged and prolonged [K^+^]_o_ undershoot in the absence of FMRP, suggesting compromised activity-dependent K^+^ clearance. Our findings are in accordance with prominent undershoot observed in the hippocampus of glial conditional Kir4.1^-/-^ mice^44^. Astroglial Na^+^/K^+^ ATPases also contribute to K^+^ clearance in the hippocampus^45^. It is noteworthy that in Kir4.1^-/-^ mice, the depolarization of astrocytes may impair the activity of Na^+^/K^+^ ATPases, therefore masking their contribution to K^+^ clearance. Indeed, as the activity of Na^+^/K^+^-ATPases is dependent on [K^+^]_o_ and [Na^+^]_i_, alteration in homeostasis of these ions is likely to alter the uptake of excess K^+^ by Na^+^/K^+^ ATPases. Thus, although we show the important role of Kir4.1 deficit in the altered extracellular K^+^ homeostasis from *Fmr1* KO mice using genetic tools altering Kir4.1 expression (Kir4.1^-/-^ mice and viral vectors to rescue Kir4.1 function), deciphering the contribution of Na^+^/K^+^ ATPases in activity-dependent K^+^ buffering in physiological conditions is complex.

Importantly, we found that FMRP associates with Kir4.1 mRNA and regulates Kir4.1 expression. This reduction in Kir4.1 levels did not result from changes in the morphology of astrocytes, which we found to be unaltered in *Fmr1* KO mice, in accordance with other studies^24,27^. It is noteworthy that Kir4.1 protein levels are also under the control of Methyl-CpG-binding protein 2 (MeCP2), a transcriptional regulator expressed in both neurons and glial cells^46^, and whose mutations lead to 95% of Rett syndrome cases^47^. MeCP2 interacts with the promoter of the KCNJ10 gene encoding Kir4.1^48^ and typical upregulation of Kir4.1 at the early postnatal development stage^36,49^ is absent in MeCP2 KO mice, suggesting altered astrocyte maturation in Rett syndrome^48^. Our findings suggest that dysregulation of Kir4.1 expression and function in the absence of FMRP is responsible for impaired [K^+^]_o_ regulation in FXS.

Previous findings show that Kir4.1 is expressed in the proximity of neuronal soma in several brain regions including lateral habenula^50^, ventral spinal cord^51^ and brainstem^52^. We observed similar pattern of perineuronal Kir4.1 staining in the hypothalamus and somatosensory cortex. However, in the CA1 pyramidal layer, only Kir4.1-positive fibers are observed^53^ while Kir4.1 expression in the *stratum radiatum* is the most intense in GFAP-positive astrocytes^50,53^. Processes of CA1 *stratum radiatum* astrocytes contact synapse sites^54^ and are therefore well-suited for the uptake of K^+^ released by dendrites of pyramidal neurons^48^ and maintenance of [K^+^]_o_ homeostasis. Downregulation and dysfunction of Kir4.1 in s*tratum radiatum* astrocytes of *Fmr1* KO male mice lead to deregulation of the K^+^ clearance following synaptic activity and thus contribute to synaptopathy in FXS. Interestingly, pronounced downregulation and reduced activity of astrocyte Kir4.1 channels have also been reported in brain tissues from epileptic patients, and in animal models of epilepsy^55^. Compromised properties of astrocyte Kir4.1 channels have been proposed to facilitate development of seizures and promote epileptogenesis^35,55,56^. This is consistent with the high incidence of seizures reported in FXS patients, and in the *Fmr1* KO mice^7,17^, supporting a key role for dysfunction of the Kir4.1 channel in mechanisms associated with epilepsy.

Increased neuronal excitability in a variety of brain regions is a prominent feature of FXS^17^. Multiple lines of evidence have linked changes in neuronal excitability in FXS to changes in expression and activity of neuronal ion channels, such as BK or Ca_V_2.2 channels^11,12,15,16^. The altered extracellular K^+^ homeostasis, resulting from reduced astroglial Kir4.1-mediated K^+^ uptake, may also contribute to the deregulation of these neuronal ion channels in FXS. Increased extracellular K^+^ levels, by inducing neuronal depolarization, can indeed regulate the activity of these neuronal ion channels^57,58^. In the present study, we discover that loss of FMRP specifically from hippocampal astrocytes causes hyperexcitability of CA1 pyramidal neurons. Moreover, we uncover dysfunction of astroglial Kir4.1 channels as a major cause of increased excitability in CA1 pyramidal neurons in FXS. Our experiments restoring astroglial Kir4.1 to WT level by viral-mediated delivery indeed rescued normal astrocyte K^+^ uptake and neuronal excitability in *Fmr1* KO hippocampus. Our data suggest that changes in Kir4.1 expression and activity in FXS have a stronger impact on high regimes of neuronal activity, since we did not observe significant [K^+^]_o_ changes in basal conditions in the *Fmr1* KO hippocampus. This is supported by a modeling study showing that astrocyte Kir4.1 channels have a pronounced effect on neuronal excitability during repetitive stimulation and a firing regime at 3-10 Hz^59^.

Neuronal hyperexcitability has been associated with several ASD-linked behavioral phenotypes. Specifically, excitatory neurons lacking cyclin-dependent kinase-like 5 display increased excitability that is associated with impaired hippocampal-dependent memory^60^. Moreover, in a rat model of autism induced by valproic acid-exposure, altered social behavior was observed together with intrinsic neuronal hyperexcitability^61^. Here, we show that astrocyte Kir4.1 channel in the hippocampus has an important role in the regulation of neuronal excitability as well as cognitive and social recognition. Selective delivery of Kir4.1 in hippocampal astrocytes rescued spatial recognition memory, as demonstrated by NOR experiments in *Fmr1* KO male mice. Moreover, this also restored the ability of *Fmr1* KO male mice to distinguish between a familiar and a novel stranger mouse, demonstrating an improvement of social recognition. Restoring Kir4.1 expression in ∼70% of hippocampal CA1 *stratum radiatum* astrocytes was sufficient to correct neuronal hyperexcitability and behavioral defects in *Fmr1* KO mice. The expression of Kir4.1 is also decreased in the somatosensory cortex of *Fmr1* KO mice. This could be associated with neuronal hyperexcitability in this brain region as well as sensory hypersensitivity^16^, which can contribute to abnormal social behavior^62,63^. Altogether, our findings support the notion that impaired astrocyte physiology in the absence of FMRP contributes to neuronal defects and behavioral abnormalities. These data point to astroglial Kir4.1 channel as a potential therapeutic target to treat FXS behavioral defects, and especially hippocampal-dependent memory impairments.

Impaired properties of Kir4.1 channel have been reported in several neurodegenerative diseases including Huntington’s disease^64^ and amyotrophic lateral sclerosis^51,65^. Emerging data point to the Kir4.1 channel as an important player in the pathophysiology of various disorders linked to ASD and intellectual disability. Mutations in the KCNJ10 gene encoding Kir4.1 have been reported to cause SeSAME/EAST syndrome characterized by early onset seizures, ataxia and mental retardation^66^. Likewise, two mutations resulting in Kir4.1 gain of function were identified in patients with ASD showing symptoms of epilepsy^67^. Recently, a KCNJ10 gene polymorphism has been correlated with ASD susceptibility^68^. Furthermore, the same study provided evidence of reduced expression of Kir4.1 in the hippocampus of the valproic acid-exposed rat model of ASD, suggesting the involvement of this astrocyte channel^68^. The KCNJ10 gene promoter is also a binding target of the MeCP2 protein implicated in Rett syndrome^48^. Mice lacking MeCP2 display reduced astroglial Kir4.1 expression and activity that is associated with increased extracellular K^+^ levels^48^. However, it is unknown whether the compromised Kir4.1 channels lead to neuronal dysfunctions in Rett syndrome. Here, we not only uncover dysfunction of Kir4.1 channels in FXS, but also reveal that this underlies several neuronal and behavioral deficits in FXS. Altogether, these data suggest that alteration in astroglial Kir4.1 channels may be involved in the pathophysiology of several neurodevelopmental disorders.

Up to now, most research has focused on neuronal cell autonomous mechanisms to account for neuronal hyperexcitability and behavioral defects in FXS. By focusing on the role of astrocytes in FXS, we here provide compelling evidence for Kir4.1 dysfunction as a major contributor to impaired extracellular K^+^ homeostasis, neuronal hyperexcitability and behavioral deficits. Herein, we propose that restoring Kir4.1 function in the hippocampus of FXS patients could be a promising therapeutic strategy for the treatment of neuronal hyperexcitability and cognitive defects.

## Methods

### Animals

All procedures on animals strictly followed the guidelines of the European Community Council Directives of January 1^st^ 2013 (2010/63/EU) and French ethic committee (certificate A751901. delivered by the French Ministry of higher education, research and innovation). All the experiments were carried out using male mice of wild-type (WT) C57BL/6j background, *Fmr1* knockout (KO) mice^5^, mice containing Cre-excisable loxP sequence in the *Fmr1* gene (*Fmr1*^fl/fl^)^69^, mice containing Cre-excisable loxP sequence in the gene encoding Kir4.1 (*Kir4.1*^*fl/fl*^) and mice with conditional deletion of Kir4.1 in glia, *Kir4.1*^*fl/fl*^:hGFAP-Cre (Kir4.1^−/−^) mice (both provided by K. D. McCarthy, University of North Carolina, USA)^35,44^. Both non-transgenic littermate and age-matched WT males were used as control mice. All animals were 21- to 32-day-old males unless otherwise stated. Adult mice were used for behavior experiments at the age of 4–5 months. Breeding and genotyping of mice were performed as previously described^70^. Mice were housed under 12 h/12 h light/dark cycle, temperature of 22 °C, humidity 55% and with food and water available *ad libitum*. All efforts were made to minimize the number of animals used.

### Preparation of hippocampal brain slices

Male WT, *Fmr1* KO, *Fmr1*^fl/fl^, *Kir4.1*^*fl/fl*^ or Kir4.1^−/−^ mice (P21-32) underwent cervical dislocation and were decapitated. The brain was isolated and the hippocampus dissected out into ice-cold artificial cerebrospinal fluid (ACSF) containing (mM): 119 NaCl, 2.5 KCl, 2.5 CaCl_2_, 1.3 MgSO_4_, 1 NaH_2_PO_4_, 26.2 NaHCO_3_ and 11 glucose continuously bubbled with a mixture of 95% O_2_ and 5% CO_2_. Acute transverse hippocampal slices (350 µm) were sectioned in cold ACSF using vibratome (Leica VT 1200S) and then recovered in ACSF at room temperature for 1 h. After recovering, slices were transferred to a recording chamber mounted on an Olympus BX51WI microscope equipped with infrared-differential interference (IR-DIC) microscopy. Slices were perfused with the oxygenated ACSF at the constant flow rate of 2 ml/min at room temperature. All chemicals and products in this study were purchased from Sigma Aldrich unless otherwise stated.

### Measurement of extracellular K^+^ concentration

K^+^-sensitive microelectrodes were made of thin wall borosilicate capillaries (GC150T-7.5, Harvard Apparatus, MA) and the interior surface was silanized during 15 min with N,N-Dimethyltrimethylsilylamine (226289, Sigma Aldrich). The electrodes were dried in the oven at 200 °C for 100 min. The tip of the electrode was filled with Potassium ionophore I—Cocktail A (60031, Sigma Aldrich) while the rest was backfilled with 200 mM KCl in ACSF. The calibration of K^+^-sensitive microelectrodes was done in the standard solutions of the following KCl concentration (mM): 0.5, 1, 2, 4, 8, 10 and 20. The extracellular potassium concentration ([K^+^]_o_) was measured in acute hippocampal slices of WT and *Fmr1* KO mice. The experiments were performed in oxygenated ACSF in the presence of 100 µM picrotoxin (P1675, Sigma Aldrich). A cut between CA1 and CA3 was made to prevent propagation of epileptiform activity. A glass pipette filled with ACSF was used to stimulate Schaffer collaterals (SC) and evoke synaptic activity in CA1 area. Extracellular field recording electrode (filled with ACSF) and K^+^-sensitive microelectrode were placed in CA1 *stratum radiatum* in close proximity (<10 µm). Basal [K^+^]_o_ was measured after correct positioning of the K^+^-sensitive microelectrode in the hippocampal slice and stabilization of the baseline. Data were acquired in current-clamp mode using Axopatch 1D and 200B amplifier, sampled at 10 kHz, filtered at 2 kHz and digitized using Digidata 1320 and 1440 (Molecular Devices). Stimulation intensity was adjusted by applying 0.05 Hz stimulation and inducing similar fiber volleys in WT and *Fmr1* KO mice. [K^+^]_o_ and fEPSPs were simultaneously recorded in response to 10 Hz train stimulus over 30 s and further analysis was performed as previously described^44^. Briefly, the signal obtained using the K^+^-sensitive microelectrode was corrected by subtracting fEPSP response. The resulting trace was linearized and converted into [K^+^]_o_ values using parameters of the log-linear fit function. The peak amplitude and area of [K^+^]_o_ transient as well as of [K^+^]_o_ undershoot were measured. The return time of [K^+^]_o_ after the train stimulus was determined as the time of baseline recovery following the undershoot using exponential fitting^44^.

### Electrophysiological recordings from astrocytes and neurons

We applied a protocol^71^ to simultaneously perform extracellular field recording and whole-cell patch-clamp on astrocytes in acute hippocampal slices of WT and *Fmr1* KO mice. The experiments were done in the presence of 100 µM picrotoxin (P1675, Sigma Aldrich) and a cut was made between CA1 and CA3 to prevent propagation of epileptiform activity. Pipettes were pulled from borosilicate glass capillaries (outer diameter 1.5 mm, inner diameter 0.86 mm, GC150F-10, Harvard Apparatus). A glass pipette filled with ACSF (300–700 kΩ) was used to electrically stimulate (0.05 Hz) Schaffer collaterals to evoke postsynaptic and astrocyte response. Stimulation intensity was adjusted to induce similar fiber volley amplitudes in WT and *Fmr1* KO hippocampal slices. fEPSP was recorded using ACSF-filled glass pipette (4–6 MΩ) placed in *stratum radiatum*. Astrocytes in *stratum radiatum* were morphologically identified according to oval shape and small cell body (5–10 µm). Whole-cell patch-clamp was performed using patch pipettes (4–6 MΩ) filled with intracellular solution containing (mM): 105 K-gluconate, 30 KCl, 10 HEPES, 10 phosphocreatine, 4 Mg-ATP, 0.3 GTP-Tris and 0.3 EGTA (pH 7.4, 280 mOsm). Resting membrane potential and membrane resistance were measured directly from the amplifier. Whole-cell currents were evoked by 150 ms voltage steps from −200 up to +40 mV in 10 mV increments. Astrocytes were distinguished according to the following electrophysiological properties: resting membrane potential around −80 mV, low membrane resistance, absence of APs and passive membrane properties represented by linear profile of I-V plot. Total synaptically-evoked astrocyte current was recorded in voltage-clamp mode by holding membrane potential at −80 mV. Addition of 10 µM (RS)-3-(2-carboxypiperazin-4-yl)-propyl-1-phosphonic acid (CPP, 0173, Tocris) and 10 µM 2,3-dioxo-6-nitro-1,2,3,4-tetrahydrobenzoquinoxaline-7-sulfonamide (NBQX, 0373, Tocris), NMDA and AMPA receptor antagonists, respectively, blocked postsynaptic activity and astrocyte potassium current (I_K_)^37^. Astrocyte I_K_ was isolated by subtracting CPP + NBQX-insensitive component from total current. The following parameters were measured to describe astrocyte I_K_ properties in WT and *Fmr1* KO mice: peak amplitude, peak amplitude normalized to fEPSP slope, charge (calculated as area under the trace), time of peak, rise time, and decay time.

Whole-cell patch-clamp was performed on CA1 pyramidal neurons in acute hippocampal slices. The experiments were performed in the presence of 100 µM picrotoxin, 10 µM CPP and 10 µM NBQX to completely block synaptic responses of the recorded cells. Pipettes were filled with intracellular solution (mM): 105 K-gluconate, 30 KCl, 10 HEPES, 10 phosphocreatine, 4 Mg-ATP, 0.3 GTP-Tris and 0.3 EGTA having pH 7.4 and osmolarity 280 mOsm. The electrophysiological properties of the pyramidal neurons were investigated in current-clamp mode. Cell membrane potential was measured 5 min after establishing whole-cell patch-clamp configuration. To ensure stability of the recording, cell membrane potential was set at −60 mV. Rheobase was determined as the minimum current required to trigger action potential (AP) by applying slow depolarization ramp protocol. AP firing properties were explored by applying depolarizing current steps of 500 ms duration from −100 pA up to 150 pA in 10 pA increments. Recordings were acquired using Axopatch-1D amplifiers (Molecular Devices) and pClamp9 software (Molecular Devices), digitized at 10 kHz using Digidata 1322 (Molecular Devices) and low-pass filtered at 2 kHz and analyzed using Clampfit10 software (Molecular Devices).

When performing dye-coupling experiments, a patch pipette was filled with intracellular solution containing biocytin (7 mg/ml, B4261, Sigma-Aldrich). Intercellular diffusion of biocytin from single patched astrocytes was performed in current-clamp mode during 20 min. The slices were subsequently fixed in 4% PFA at 4 °C overnight and further stained using Alexa-488 conjugated streptavidin (1:300, S11223, Invitrogen). Confocal images were acquired and the number of coupled cells was determined using ImageJ software (National Institute of Health, USA).

For astrocyte morphology analysis, patch pipettes contained 175 µM Alexa-488 hydrazide (A10436, Invitrogen) diluted in intracellular solution. The astrocytes were loaded with Alexa-488 dye in current clamp mode for 20 min and then fixed overnight in 4% PFA at 4 °C. Confocal images of loaded astrocytes were acquired using x63 objective and adjusting z-step to 0.3 µm. After image deconvolution using Huygens software (Scientific Volume Imaging), morphological reconstruction of each cell was performed in Imaris software (OXFORD Instruments). Sholl analysis was performed to count the number of intersections between astrocyte processes and circles at increasing distances from the cell center.

### Immunoprecipitation and RT-PCR

For the immunoprecipitation protocol^72^, hippocampi from WT and *Fmr1* KO mice (P28-32) were homogenized in the ice-cold buffer (500 µl per mouse) containing: 10 mM HEPES pH 7.4, 150 mM KCl, 5 mM MgCl_2_, 0.5 mM DTT, 40 U/ml RNaseOUT (10777019, Invitrogen) and 0.1 µg/ml cycloheximide (CHX) supplemented with protease inhibitors (Sigma-Aldrich). The homogenates were clarified by centrifugation at 2000 × *g* for 10 min at 4 °C, supernatant was collected and 10% NP-40 and 1,2-diheptanoyl-sn-glycero-3-phosphocholine (DHPC, 850306, Avanti) were added to complete the lysis. Protein extracts were spun at 16,000 × *g* for 15 min at 4 °C. The resulting supernatants were pre-cleared for 30 min at 4 °C with 200 µl of magnetic beads, Dynabeads^TM^ Protein G (1003D, Invitrogen). The samples were then incubated with Dynabeads coupled to either 15 µg of monoclonal mouse anti-FMRP antibody (7G1-1, Developmental Studies Hybridoma Bank) or 5 µg of anti-mouse IgG (ab37355, Abcam) for 30 min at 4 °C. The monoclonal antibody 7G1–1 developed by Stephen T. Warren was obtained from the Developmental Studies Hybridoma Bank, created by the NICHD of the NIH and maintained at the University of Iowa, Department of Biological Sciences, Iowa City IA 55242. Beads were washed three times in high-salt buffer containing: 10 mM HEPES pH 7.4, 350 mM KCl, 5 mM MgCl_2_, 0.5 mM DTT, 1% NP-40, 40 U/ml RNaseOUT and 0.1 µg/ml CHX. After the last wash, samples were resuspended in RTL buffer and RNA was isolated from the IP complex using RNeasy lipid tissue mini kit (74804, Qiagen) according to the producer’s instructions. Extracted RNA was then converted to cDNA using SuperScript III Reverse Transcriptase (18080093, Invitrogen) and the RT-PCR was performed using primers specific for Kir4.1 (Forward: 5’-TGGTGTGGTGTGGTATCTGG-3’, Reverse: 5’-TGAAGCAGTTTGCCTGTCAC-3’), PSD-95 (positive control; Forward: 5’-GGCTTCATTCCCAGCAAACG-3’, Reverse: 5’-CATCAAGGATGCAGTGCTTC-3’)^39^ and GLT-1 mRNAs (negative control; Forward: 5’-ACAATATGCCCAAGCAGGTAGA-3’, Reverse: 5’-CTTTGGCTCATCGGAGCTGA-3’)^24^.

### Immunohistochemistry and image analysis

WT and *Fmr1* KO mice (P27-32) were anesthetized with lethal dose of Dolethal (150 µl/10 g) and transcardially perfused with 4% paraformaldehyde (PFA). Brains were removed, postfixed in 4% PFA for 8 h and dehydrated in 30% sucrose at +4 °C. Free-floating coronal sections (40 µm) were cut on a microtome, collected in phosphate-buffer saline (PBS) and stored at +4 °C for further use. After several washes in PBS, sections were heated for 30 min in antigen retrieval solution: 10 mM citrate buffer containing 0.05% Tween20 (pH 6). After cooling at room temperature, slices were placed in a blocking solution comprising 10% normal goat serum (NGS) and 0.5% Triton X-100 for 1 h at room temperature. Primary antibody incubation was carried out overnight at +4 °C using monoclonal mouse anti-GFAP (1:300, clone G-A-5, G3893, Sigma Aldrich), rabbit anti-Kir4.1 (1:100, APC-035, Alomone Labs) and polyclonal chicken anti-GFP (1:500, AB13970, Abcam) in 2% NGS, 0.5% Triton X-100. Several washes in PBS were followed by incubation with secondary antibodies: goat anti-mouse IgG conjugated to Alexa 488 (1:1000, A11029, Life Technologies), goat anti-rabbit IgG conjugated to Alexa 555 (1:1000, A21424, Life Technologies) and goat anti-chicken IgG conjugated to Alexa 488 (1:1000, A11039, Life Technologies) diluted in 2% NGS for 2.5 h at room temperature. Nuclei were labeled using 4′,6-Diamidino-2-phenylindole dihydrochloride (DAPI; 1:2000, D9542, Sigma Aldrich). After rinsing several times, slices were mounted on microscope slides using Fluoromount-G (SouthernBiotech). All antibodies used in this study are commercially available and were validated by manufacturer and/or studies cited by the company’s website.

Immunofluorescence was visualized with the inverted confocal microscopes (TCS SP5, Leica and Zeiss LSM 800) using x63 oil immersion objective and images were acquired using LAS X (Leica) or Zeiss ZEN software. When acquiring/analyzing the immunohistochemistry data, the investigators were not blind to the genotype. Images of CA1 *stratum radiatum* were acquired as z-stacks of 15–18 slices at a distance of 1 µm. Several fields of view of CA1 hippocampal region were captured per slice. Z-projections were reconstructed using ImageJ software, *stratum radiatum* was outlined to select the region of interest (ROI) and Kir4.1 signal intensity was quantified as integrated density. GFAP integrated density was measured in the same ROI and then Kir4.1/GFAP integrated densities ratio was calculated. Astrocyte domain was outlined based on GFAP or GFP staining when Kir4.1 integrated density was quantified per single astrocyte after AAV injections. To calculate distribution of Kir4.1 intensity, we used the Radial profile plugin in ImageJ and measured normalized integrated density along concentric circles as a function of radial distance from the soma center.

### Fluorescent in situ hybridization (FISH)

WT mice (P30-32) were anesthetized with lethal dose of Dolethal (150 µl/10 g) and transcardially perfused with 4% PFA, brain was isolated and frozen brain sections were prepared. After washing slices in PBS, they were incubated with 7 drops of RNAscope hydrogen peroxide for 10 min at RT, then rinsed in 50 mM Tris-buffered saline with 0.1% Tween20 and mounted on microscope slides. Slices were dried for 1 h in the dark at RT, then shortly submerged in deionized water and dried again for additional 1 h in the dark at RT. After incubation of slices for 1 h at 60 °C in a dry oven, they were dried overnight in the dark at RT. After short immersion of slices into deionized water, they were incubated for a few seconds with the drop of ethanol and then incubated in a steamer at 100 °C. A drop of RNAscope 1x Target Retrieval Reagent was added to the steamer for 15 min incubation. Slices were rinsed in deionized water before adding a drop of ethanol for 3 min and then a drop of RNAscope Protease+ solution for an incubation in humid box at 40 °C for 30 min and washed three times in deionized water. FISH protocol was performed following v2 Multiplex RNAscope technique (Advanced Cell Diagnostics, Inc., Newark, CA, USA)^73^. We used RNAscope® probe Mm_Kcnj10_C2 (Bio-techne) to label Kir4.1 mRNA. Following the FISH procedure, slices were incubated with a solution of primary antibody, monoclonal mouse anti-FMRP (1:100, 7G1-1, Developmental Studies Hybridoma Bank) in 5% normal goat serum and 0.1% Triton-X 100 overnight at 4 °C. Slices were rinsed three times in PBS and incubated with the secondary antibody goat anti-mouse IgG conjugated to Alexa 555 (1:200, A21424, Life Technologies) for 2.5 h at RT. After washing in PBS, the slides were mounted in Fluoromount-G® (Southern Biotech) and image acquisition was performed using confocal microscope Zeiss LSM 800.

### Surface biotinylation and Western blot

Acute hippocampal slices from WT and *Fmr1* KO mice (P25-32) were stored at RT in ACSF for 1 h and then transferred in the solution containing 1 mg/ml biotin (EZ-Link® Sulpho-NHS-SS-Biotin, 21331, Thermo Scientific)^74^ and incubated for 45 min on ice. Slices were washed three times in ice cold quench buffer containing 100 mM glycine in ACSF and incubated two times 25 min in the same buffer to quench unbound sulfo-NHS-SS-biotin. The slices were homogenized in the RIPA buffer containing: 100 mM Tris, 150 mM NaCl, 1 mM EDTA, 1% Triton-1x-100, 0.1% SDS and 1% Na deoxycholate supplemented with protease inhibitors cocktail and incubated for 30 min at 4 °C to complete the lysis. After centrifugation at 16,000 × *g* for 15 min at 4 °C, supernatant was taken and frozen at −80 °C until further use. Protein concentration was determined using Ionic Detergent Compatibility Reagent (22663, Thermofisher, France). 60 µg of each sample was incubated with 20 µl of precleared streptavidin agarose beads (Pierce™ Streptavidin Plus UltraLink™ Resin, 53116, Themo Scientific) overnight at +4 °C. After centrifugation at 16,000 × *g* for 2 min at RT, the beads were washed three times in RIPA buffer. Finally, 30 µl of 2× Laemli buffer was added to each sample and rotated for 30 min at RT and then spun down. 15 µl of each biotinylated sample was loaded on precast 4%–12% gradient gel (NuPAGE Novex Bis-Tris gel, NP0321BOX, Invitrogen). The proteins were transferred onto nitrocellulose membrane and saturated with 5% fat-free dried milk in triphosphate buffer solution. Membranes were incubated with primary antibody rabbit anti-Kir4.1 (1:1000, APC-035, Alomone Labs) at 4 °C overnight. On the next day, membranes were incubated with horseradish peroxidase (HRP)-conjugated secondary antibody goat anti-rabbit-HRP (1:2000, CSA2115, Cohesion Biosciences) for 2 h at RT. Monoclonal mouse anti-β actin-HRP antibody (1:2000, clone AC-15, ab49900, Abcam) was used for loading controls. Membranes were revealed using a chemiluminescence detection kit (ECL, 28980926, GE Healthcare) and visualized using ImageQuant LAS 4000 imaging system (GE Healthcare) and ImageQuant LAS 4000 software (Fujifilm). Uncropped and unprocessed scans of the blots are supplied in the Source Data file.

### Recombinant adeno-associated virus (rAAV) generation and stereotaxic microinjections

For rAAV in vivo gene transfer, a transgene composed of GFP, Cre recombinase and Kir4.1 cDNA separated by P2A sequence in a single open reading frame was placed under the control of a GFAP-specific promoter (gfaABC_1_D) in a rAAV shuttle plasmid containing the inverted terminal repeats (ITR) of AAV2 (AAV-gfaABC_1_D-Kir4.1-GFP)^64^. Pseudotyped serotype 5 or 9 rAAV particles were produced by transient co-transfection of HEK-293T cells^75^. Viral titers were determined by quantitative PCR amplification of ITR on DNase-resistant particles and expressed as vector genome per ml (vg/ml). *Fmr1* KO, *Fmr1*^fl/fl^, *Kir4.1*^*fl/fl*^ or WT mice were deeply anesthetized using a mixture of ketamine (95 mg/kg; Merial) and xylazine (10 mg/kg; Bayer) in 0.9% NaCl and placed into a stereotaxic frame (David Kopf Instruments). Unilateral injections into the hippocampus of WT, Fmr1 KO, *Fmr1*^fl/fl^ or *Kir4.1*^*fl/fl*^ young mice (P15–17) were carried out using the following coordinates from Bregma: antero-posterior −1.94 mm, medio-lateral −1.5 mm and dorso-ventral −1.38 mm from the brain surface. 0.5 µl of AAV2/5-gfaABC_1_D-Kir4.1-GFP or AAV2/5-gfaABC_1_D-GFP (2.5 × 10^13^ vg/ml) or AAV2/9-gfaABC_1_D-Cre-GFP (10^13^ vg/ml) was injected at the rate of 0.05 µl/min. Bilateral injections into the hippocampus of WT, *Fmr1* KO or *Kir4.1*^*fl/fl*^ adult mice (~3-month-old) were performed using coordinates from Bregma as follows: antero-posterior −2.0 mm, medio-lateral ±1.5 mm and dorso-ventral −1.5 mm. 1 µl of AAV2/5-gfaABC_1_D-Kir4.1-GFP or AAV2/5-gfaABC_1_D-GFP (2.5 × 10^13^ vg/ml) or AAV2/9-gfaABC_1_D-Cre-GFP (10^13^ vg/ml) was injected per each side of the brain at the rate of 0.2 µl/min. The injections were performed using 29-gauge blunt-tip needle connected to 2 µl Hamilton syringe and the injection rate was controlled by syringe pump (KD Scientific). Following microinjection, the needle was left in place for 5 min prior to slow withdrawal. After surgery, mice were recovered from anesthesia on a heating pad and monitored for the next 24 h. When AAV2 was injected unilaterally into the hippocampus of young mice P15–17, they were sacrificed two weeks post injection for electrophysiology and immunohistochemistry experiments. In case of bilateral AAV2 injection into the hippocampus of 3 month-old adult mice, they were used for behavioral testing 1-2 months post injection.

### Behavioral testing

The behavioral assays were conducted in The Experimental and Molecular Immunology and Neurogenetics laboratory in Orléans, France. Mice were examined in behavioral assays 1–2 months after bilateral injection of AAV2/5 GFP into the hippocampus of WT and *Fmr1* KO mice, AAV2/5 Kir4.1-GFP into the hippocampus of *Fmr1* KO mice or AAV2/9 Cre-GFP into the hippocampus of WT and *Kir4.1*^*fl/fl *^ mice. All behavioral tests were carried out during the light phase of the cycle and the experimental set up was cleaned with 70% ethanol after each session and before investigating another mouse. Prior to behavioral testing, animals were placed into the experimental room and allowed to acclimatize for 30 min. A numeric camera was used for recording of mice while performing the test and the obtained data were analyzed by PC-based video tracking software (EthoVision XT, Noldus Technology, Netherlands).

#### Novel object recognition (NOR) test

NOR was performed in the open-field arena (48.5 cm × 48.5 cm × 29 cm) over 6 days. The first day of NOR was the habituation phase when the mouse was placed in an empty arena and allowed to freely explore the space for 10 min. On day two of the behavioral experiment, two identical objects were placed at a diagonal position inside the arena and evenly spaced from the nearest corner. The mouse was placed in the right corner of the chamber equidistant to the two objects and allowed to explore them for 10 min. This training phase was repeated under the same conditions during three subsequent days to promote familiarization with the objects. On the last day, the training was first performed and then the test mouse was removed from the chamber and placed in a single-housing cage for 2 min retention interval. Meanwhile, one of the familiar objects was replaced by a novel object different in shape, color and texture. In the testing phase, the mouse was placed in the arena in the presence of the familiar and novel objects and allowed to explore them for 10 min. The exploration time was determined as the duration of nose interaction with each object investigated during both the training and testing phase. Analysis of the exploration time was performed during the first 2 min corresponding to the pronounced exploratory period. The recognition index for each object was calculated as follows: (Time exploring novel object)/(Total time exploring both objects) × 100. When the mouse’s nose did not contact one or both objects during the training and/or testing phase, the data were excluded from the analysis.

#### Three-chamber social interaction test

This test was performed to test social interactions in mice^70,76^. The arena (25 × 50 cm) made of non-transparent plexiglass walls was used, and transparent dividers separated the arena space into three chambers of equal dimensions that were connected by a square opening (5 × 5 cm) on each divider. During the 5 min habituation phase, the mouse was placed in the central chamber and allowed to explore the arena with empty wire mesh cylinders positioned in the right and the left chamber. The next session was a 5 min sociability phase when an unfamiliar juvenile male of DBA/2j strain (Stranger 1) was placed underneath a wire mesh cylinder on one side of the arena while an inanimate object was positioned in the cylinder on the other side of arena. During the last session, the object was replaced by a novel juvenile male of DBA/2j strain (Stranger 2). The test mouse was allowed to explore the chambers during the social recognition phase for 5 min. The preference to explore the mouse versus object or the novel versus familiar mouse was evaluated. Time spent in sniffing each wire mesh cylinder containing object or mouse was measured as the duration the test mouse nose spent within 2 cm radius from the cylinder surface. The recognition index was calculated in sociability and social recognition phase as: (Sniffing time of object or mouse)/(Total sniffing time) × 100.

### Statistics

Statistical analysis was performed using SigmaPlot v11 and GraphPad Prism v6 software. Normality and equal variance between groups were performed before statistical comparison and the appropriate parametric or non-parametric test was applied. Unpaired Student’s two-tailed *t* test or Mann–Whitney test were performed to assess the difference between two groups of data. Difference between multiple groups was evaluated using One-way ANOVA or two-way ANOVA followed by *post hoc* test. Significance was assigned at *P* < 0.05 and all data are presented as mean ± s.e.m. The sample size and the statistical test used are stated in each case in the figure legends and results.

### Reporting summary

Further information on research design is available in the Nature Portfolio Reporting Summary linked to this article.

### Supplementary information

Supplementary Information

Reporting Summary

### Source data

Source Data

## Data Availability

The data generated in this study are available in the main text and the Supplementary Information file. Source data are provided with this paper.
