# Supplementary material for: Astroglial Kir4.1 potassium channel deficit drives neuronal hyperexcitability and behavioral defects in Fragile X syndrome mouse model
Source: Nat Commun. 2024 Apr 27;15:3583. doi: 10.1038/s41467-024-47681-y (PMC11055954; doi:10.1038/s41467-024-47681-y)
Supplement: Supplementary file 1 — Supplementary Information [file 41467_2024_47681_MOESM1_ESM.pdf]

Supplementary Information for

Astroglial Kir4.1 potassium channel deficit drives neuronal hyperexcitability and behavioral defects in Fragile X syndrome

Danijela Bataveljic[1&], Helena Pivonkova[1,#], Vidian de Concini[2], Betty Hébert[2], Pascal Ezan[1], Sylvain Briault[2,3], Alexis-Pierre Bemelmans[4], Jacques Pichon[2], Arnaud Menuet[2], Nathalie Rouach[1*]

*Correspondence to: nathalie.rouach@college-de-france.fr

This PDF file includes:

Supplementary Figures 1-12

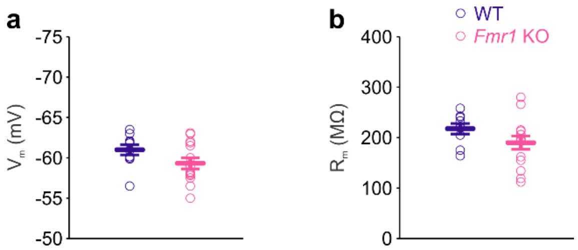

Supplementary Fig. 1. Membrane properties of CA1 pyramidal neurons are not changed in *Fmr1* KO mice. a, Resting membrane potential (V_m) and **b,** membrane resistance (R_m) remain unaltered in CA1 pyramidal cells from *Fmr1* KO mice (n=14 neurons from 14 slices in 6 mice; magenta) in comparison to pyramidal cells from WT mice (n=10 neurons from 10 slices in 6 mice; dark blue). Data are presented as mean values ± SEM (a, b). V_m: P=0.102, t=-1.709, df=22; R_m: P=0.159, t=1.460, df=22; two-sided unpaired Student's *t*-test. Source data are provided as a Source Data file.

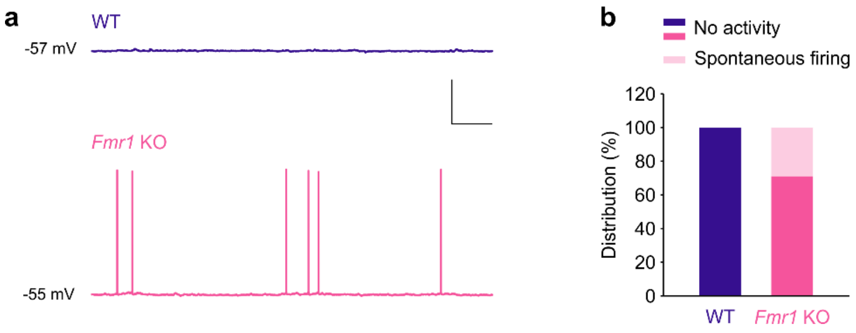

Supplementary Fig. 2. Spontaneous neuronal activity in acute hippocampal slices of *Fmr1* KO mice. a, Examples of spontaneous neuronal activity in the hippocampal CA1 area from WT (dark blue) and *Fmr1* KO mice (magenta). Scale bar: 50 mV, 50 s. **b,** Spontaneous firing was detected in *Fmr1* KO mice in 29% of the recorded pyramidal cells (n=14 from 14 slices in 6 mice) while no spontaneous activity was observed in WT neurons (n=10 from 10 slices in 6 mice). Source data are provided as a Source Data file.

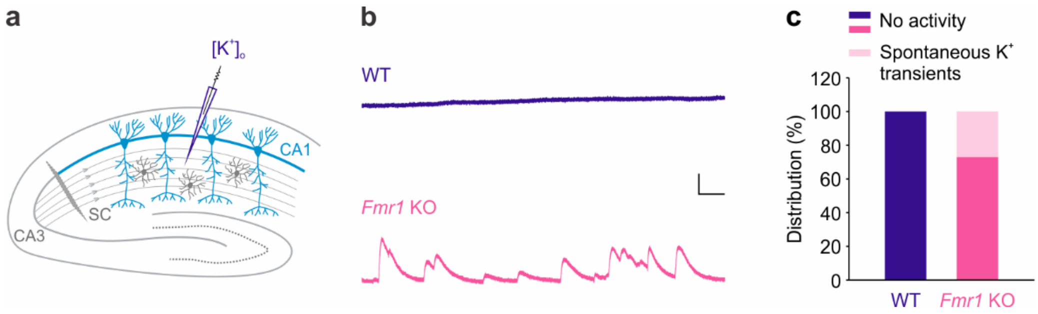

Supplementary Fig. 3. Extracellular K⁺ levels under basal conditions in *Fmr1* KO hippocampus. **a**, Hippocampus scheme illustrating position of the K^+-sensitive microelectrode placed in *stratum radiatum*. **b,** Examples of spontaneous changes in extracellular K^+ levels ($[K^+]_o$) in acute hippocampal slices from WT (dark blue) and *Fmr1* KO mice (magenta). Scale bar: 0.1 mM, 10 s. **c**, Spontaneous changes in $[K^+]_o$ were detected in ~25% of *Fmr1* KO slices (n= 8 from 7 mice), but were not observed in WT slices (n= 9 from 9 mice). Source data are provided as a Source Data file.

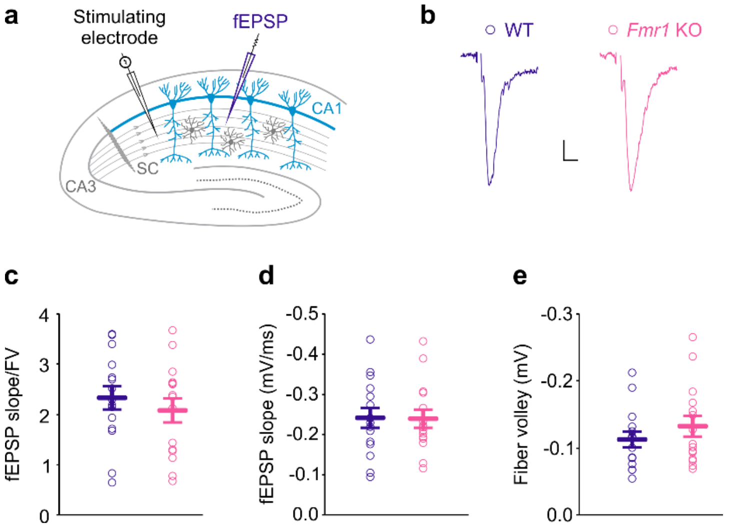

Supplementary Fig. 4. Excitatory synaptic transmission in *Fmr1* KO hippocampus. a, Schematic diagram of hippocampus depicting position of stimulating electrode that activates Schaffer collaterals (SC) and extracellular registration electrode that registers field excitatory postsynaptic potential (fEPSP). **b**, Representative examples of synaptically-evoked fEPSP responses in WT (dark blue) and *Fmr1* KO (magenta) hippocampus. Scale bar: 0.1 mV, 10 ms. **c**, Ratio of fEPSP slope and fiber volley (FV) amplitude is not changed in *Fmr1* KO (n=15 astrocytes from 15 slices in 11 mice) as compared to WT (n=15 astrocytes from 15 slices in 14 mice; P=0.460, t=0.748, df=28, two-sided unpaired Student's *t* test). **d,** fEPSP slope and **e**, FV of WT (n=15 astrocytes from 15 slices in 14 mice) and *Fmr1* KO (n=15 from 15 slices in 11 mice) are similar (fEPSP slope: P=0.950, t=-0.063, df=28, two-sided unpaired Student's *t* test; FV: P=0.407, U=92, two-sided Mann-Whitney test). Data are presented as mean values ± SEM (c-e). Source data are provided as a Source Data file.

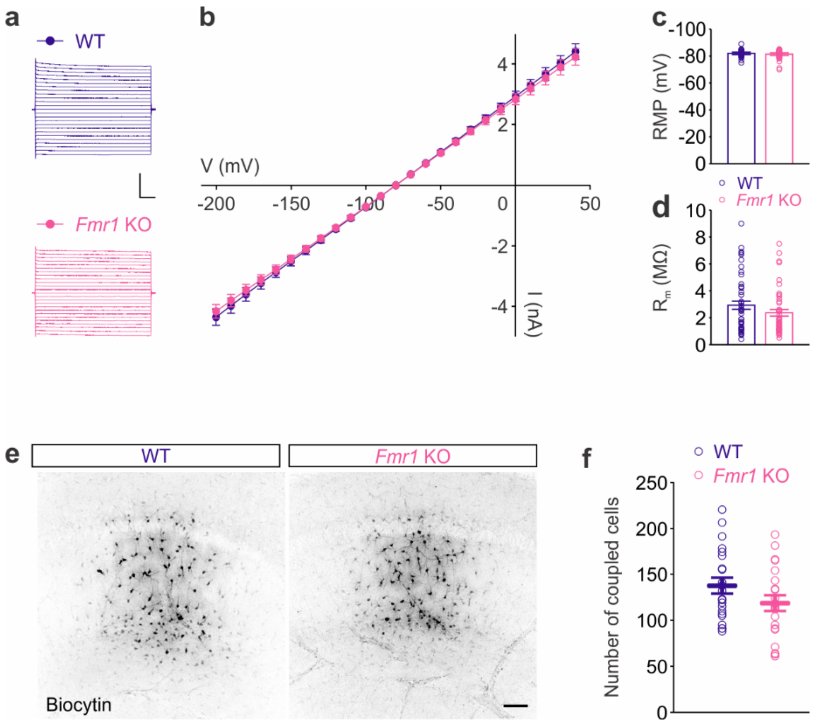

Supplementary Fig. 5. Astrocyte membrane properties and astrocyte coupling are not altered under basal conditions in *Fmr1* KO hippocampus. a, Representative current traces from WT (dark blue) and *Fmr1* KO (magenta) astrocytes evoked by voltage steps from -200 to +40 mV in 10 mV increments. Scale bar: 2 nA, 20 ms. **b,** Current-voltage (I-V) plots recorded from WT (n=48 cells from 48 slices in 29 mice) and *Fmr1* KO (n=47 cells from 47 slices in 27 mice) *stratum* radiatum astrocytes are similar (P=0.148, F(24, 1728)=1.304, two-way ANOVA repeated measures). **c,** Resting membrane potential (RMP) and **d**, membrane resistance (R_m) of *Fmr1* KO astrocytes (n=47 from 47 slices in 27 mice) are not altered when compared to WT (n=48 from 48 slices in 29 mice; RMP: P=0.690, U=1074.5; R_m: P=0.253, U=974, two-sided Mann-Whitney test). **e**, Representative confocal images of biocytin diffusion into the astrocyte network in WT and *Fmr1* KO CA1 *stratum radiatum*. Scale bar: 100 µm. **f**, Number of coupled cells following biocytin loading is unchanged in *Fmr1* KO (n=21 from 21 slices in 9 mice) as compared to WT (n=21 from 21 slices in 11 mice; P=0.125, t=1.568, df=40, two-

sided unpaired Student's *t*-test). Data are presented as mean values ± SEM (c, d, f). Source data are provided as a Source Data file.

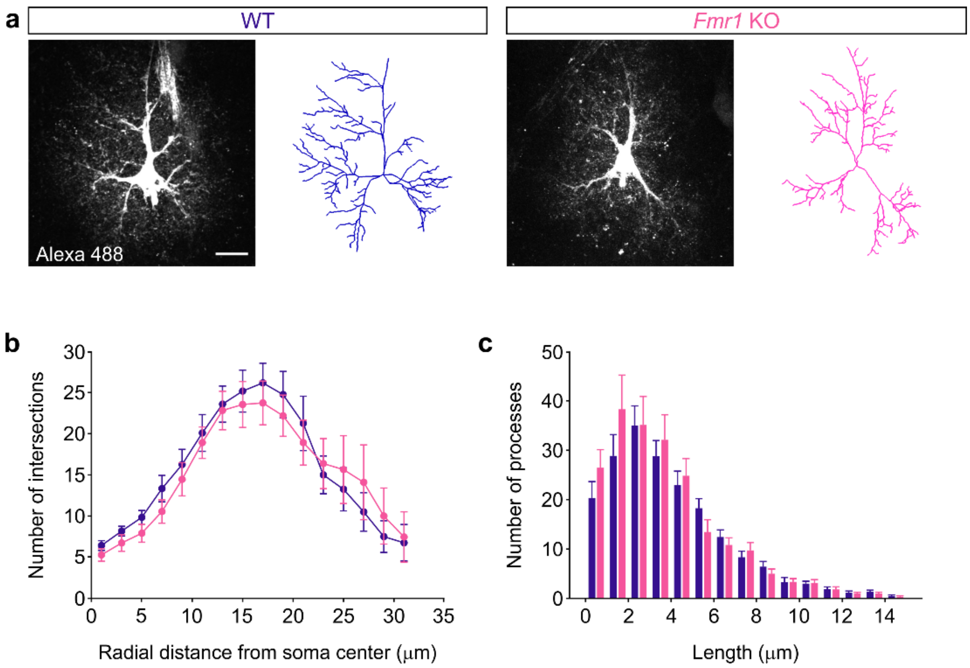

Supplementary Fig. 6. FMRP deficiency does not affect astrocyte morphology. **a,** Representative confocal images of Alexa-488 loaded astrocytes and corresponding reconstructions representing morphological charts of WT (dark blue) and *Fmr1* KO (magenta) astrocytes. Scale bar: 10 μm. **b**, Morphological Sholl analysis of Alexa-488 loaded astrocytes reveals no difference in the number of intersections at increasing radial distances from soma center between WT (n=12 cells from 12 slices in 5 mice) and *Fmr1* KO (n=11 cells from 11 slices in 7 mice) hippocampal astrocytes (P=0.989, F(32, 672)=0.5136, two-way ANOVA repeated measures). **c**, Number of astrocyte processes of increasing length is not changed in *Fmr1* KO (n=11 cells from 11 slices in 7 mice) as compared to WT (n=12 cells from 12 slices in 5 mice) mice (P=0.248, F(14, 294)=1.237 two-way ANOVA repeated measures). Data are presented as mean values ± SEM (b, c). Source data are provided as a Source Data file.

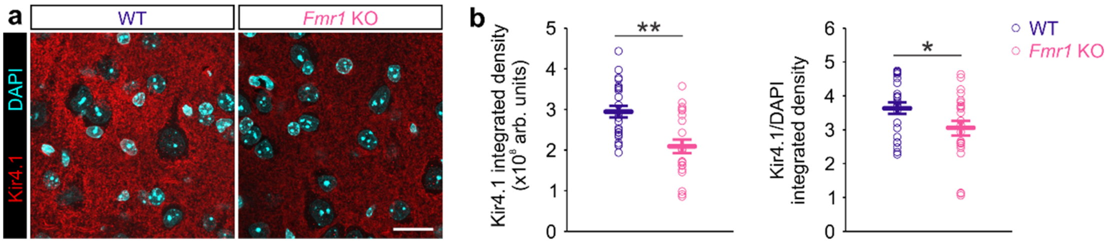

Supplementary Fig. 7. Decreased Kir4.1 expression in the somatosensory cortex of *Fmr1* KO mice. a, Confocal images of immunolabeling of Kir4.1 (red) and nucleus (DAPI, cyan) in WT (dark blue) and *Fmr1* KO (magenta) somatosensory cortex. Scale bar: 20 μm. **b,** Reduced Kir4.1 integrated density (P=0.001, U=116, two-sided Mann-Whitney test) as well as Kir4.1/DAPI integrated density (P=0.037, t=2.154, df=44, two-sided unpaired Student's t-test) in *Fmr1* KO somatosensory cortex (n=23 images from 4 mice) in comparison to WT (n=23 images from 3 mice). Data are presented as mean values ± SEM (b). Arb. units: arbitrary units. Source data are provided as a Source Data file.

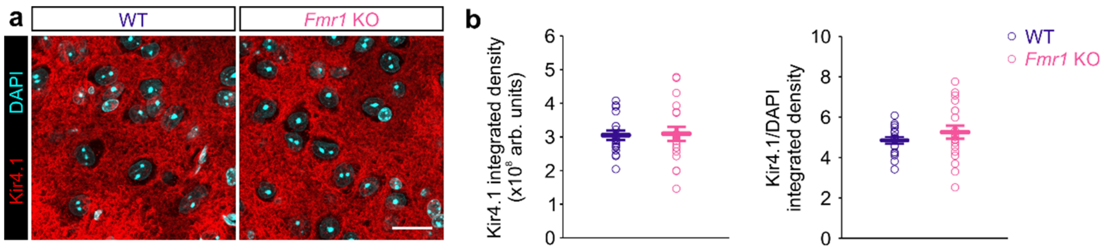

Supplementary Fig. 8. Kir4.1 expression is not altered in the hypothalamus of *Fmr1* KO mice. a, Confocal images of immunolabeling of Kir4.1 (red) and nucleus (DAPI, cyan) in WT (dark blue) and *Fmr1* KO (magenta) somatosensory cortex. Scale bar: 20 µm. **b**, Kir4.1 integrated density (P=0.857, t=-0.181, df=35, two-sided unpaired Student's t-test) as well as Kir4.1/DAPI integrated density (P=0.475, U=147, two-sided Mann-Whitney test) are not altered in *Fmr1* KO hypothalamus (n=19 images from 4 mice) in comparison to WT (n=18 images from 3 mice). Arb. units: arbitrary units. Data are presented as mean values ± SEM (b). Source data are provided as a Source Data file.

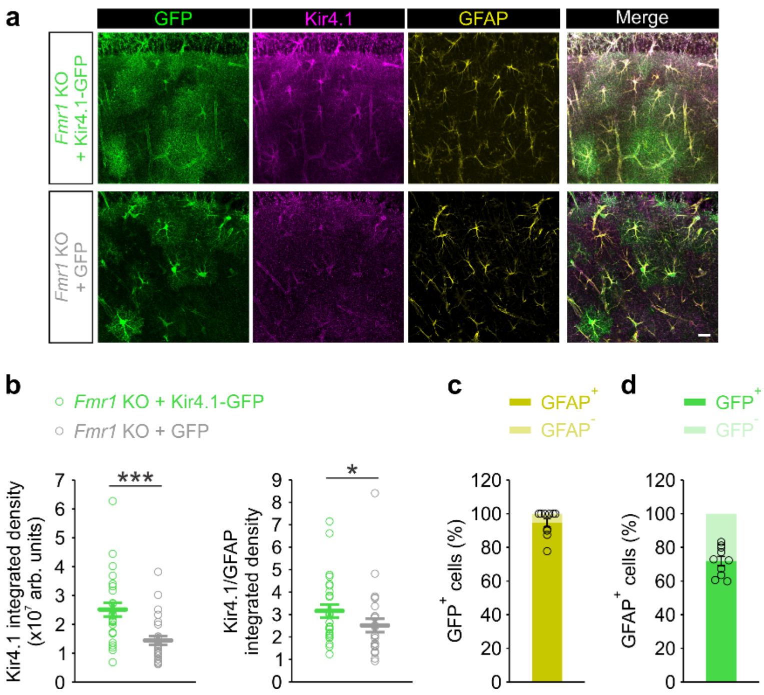

Supplementary Fig. 9. Rescue of Kir4.1 expression by AAV2/5 Kir4.1-GFP delivery into *Fmr1* KO hippocampus. a, Representative confocal images of co-immunostaining of green fluorescent protein (GFP, green), Kir4.1 (magenta) and GFAP (yellow) following Kir4.1-GFP (upper row; green) or GFP viral delivery (bottom row; grey) into hippocampus of *Fmr1* KO mice. Scale bar: 20 µm. **b**, Expression of Kir4.1 (P<0.001, U=139, two-sided Mann-Whitney test) as well as Kir4.1/GFAP ratio (P=0.033, U=231, two-sided Mann-Whitney test) in GFP-positive (GFP^+) astrocytes are significantly enhanced after transduction using Kir4.1-GFP (n=26 from 4 mice) as compared to GFP only (n=27 from 4 mice). **c**, The majority of GFP^+ cells are also $GFAP^+$ (GFP^+ $GFAP^+$: n=88 from 4 mice, dark yellow; GFP^+ $GFAP^-$: n=5 from 4 mice, light yellow). (d) Most of the $GFAP^+$ cells, ~70%, express GFP following viral transduction ($GFAP^+$ GFP^+: n=234 from 4 mice, green; $GFAP^+$ GFP^-: n=97 from 4 mice, light green). Arb. units: arbitrary units. Data are presented as mean values ± SEM (b-d). Source data are provided as a Source Data file.

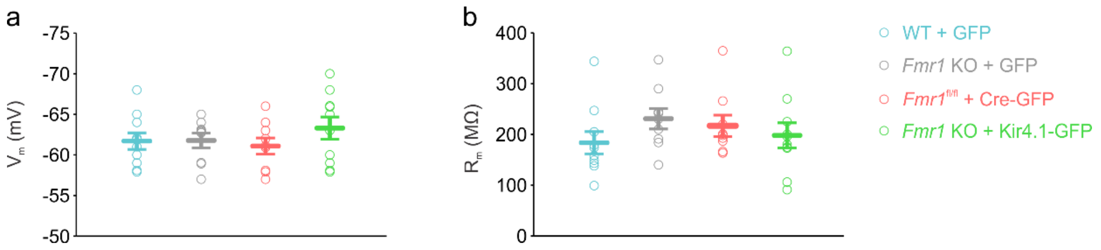

Supplementary Fig. 10. Unaltered membrane properties of CA1 pyramidal neurons following transduction with the different AAV constructs. a, Resting membrane potential (V_m) and **b**, membrane resistance (R_m) remain unaltered in *Fmr1* KO + GFP (n=9 neurons from 9 slices in 6 mice, grey), *Fmr1*$^{fl/fl}$ + Cre-GFP (n=9 neurons from 8 slices in 4 mice, red) and *Fmr1* KO + Kir-GFP (n=10 neurons from 9 slices in 6 mice, green) in comparison to WT + GFP pyramidal cells (n=10 neurons from 10 slices in 4 mice, blue). GFP: green fluorescent protein. V_m: P=0.544, F(3, 34)=0.7245 one-way ANOVA, R_m: P=0.214, H=4.479, one-way ANOVA on Ranks. Data are presented as mean values ± SEM (a, b). Source data are provided as a Source Data file.

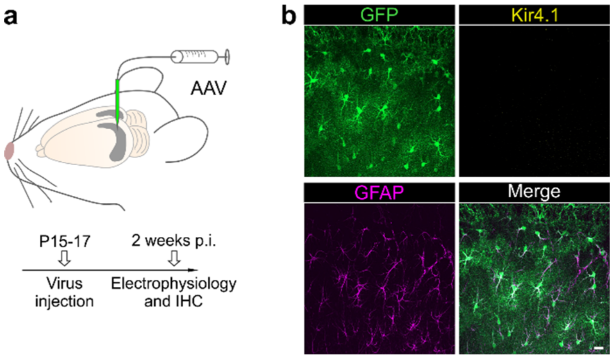

Supplementary Fig. 11. a, Illustration of adeno associated virus (AAV) bilateral injection into the hippocampus of P15-17 male mice. Electrophysiology and immunohistochemistry (IHC) were performed 2 weeks post-injection (p.i.). **b,** Representative confocal images of co-immunostaining of green fluorescent protein (GFP, green), Kir4.1 (yellow) and GFAP (magenta) following Cre-GFP viral delivery into the CA1 hippocampus of *Kir4.1*$^{fl/fl}$ mice (n=3 mice). Note the absence of Kir4.1 expression. Scale bar: 20 μm.

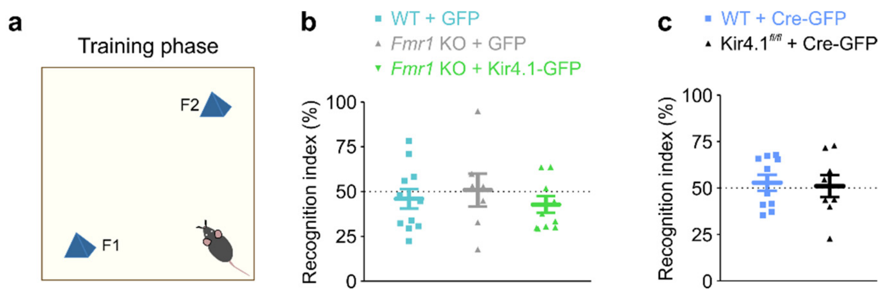

Supplementary Fig. 12. Performance of mice in the training phase of the novel object recognition test. a, Experimental set-up of the novel object recognition test in the training phase. Mice were exposed to two identical objects (F1 and F2) in the training phase, **b, c,** During the training phase, all examined mice displayed similar preference and recognition index for the two identical objects F1 and F2 (**b**) (WT+GFP mice, light blue, n=11, P=0.399, t=0.8815, df=10; *Fmr1* KO +GFP mice, grey, n=7, P=0.996, t=0.0047, df=6; *Fmr1* KO+Kir4.1-GFP mice, green, n=9, P=0.119, t=1.7470, df=8; two-sided one sample *t*-test to 50%) (**c**) (WT+Cre-GFP mice, blue, n=10, P=0.366, t=0.9517, df=9; *Kir4.1*$^{fl/fl}$+Cre-GFP mice, black, n=8, P=0.714, t=0.3822, df=7; two-sided one sample *t*-test to 50%). GFP: green fluorescent protein. Data are presented as mean values ± SEM (b, c). Source data are provided as a Source Data file.
