# Supplementary material for: Astroglial Kir4.1 potassium channel deficit drives neuronal hyperexcitability and behavioral defects in Fragile X syndrome mouse model
Source: Nat Commun. 2024 Apr 27;15:3583. doi: 10.1038/s41467-024-47681-y (PMC11055954; doi:10.1038/s41467-024-47681-y)
Supplement: Supplementary file 2 — Reporting Summary [file 41467_2024_47681_MOESM2_ESM.pdf]

nature portfolio

Corresponding author(s): Nathalie Rouach

Last updated by author(s): March 1st 2024

Reporting Summary

Nature Portfolio wishes to improve the reproducibility of the work that we publish. This form provides structure for consistency and transparency in reporting. For further information on Nature Portfolio policies, see our Editorial Policies and the Editorial Policy Checklist.

Statistics

For all statistical analyses, confirm that the following items are present in the figure legend, table legend, main text, or Methods section.

n/a	Confirmed	
☐	☒	The exact sample size (*n*) for each experimental group/condition, given as a discrete number and unit of measurement
☐	☒	A statement on whether measurements were taken from distinct samples or whether the same sample was measured repeatedly
☐	☒	The statistical test(s) used AND whether they are one- or two-sided<br>*Only common tests should be described solely by name; describe more complex techniques in the Methods section.*
☐	☒	A description of all covariates tested
☐	☒	A description of any assumptions or corrections, such as tests of normality and adjustment for multiple comparisons
☐	☒	A full description of the statistical parameters including central tendency (e.g. means) or other basic estimates (e.g. regression coefficient) AND variation (e.g. standard deviation) or associated estimates of uncertainty (e.g. confidence intervals)
☐	☒	For null hypothesis testing, the test statistic (e.g. *F*, *t*, *r*) with confidence intervals, effect sizes, degrees of freedom and *P* value noted<br>*Give P values as exact values whenever suitable.*
☒	☐	For Bayesian analysis, information on the choice of priors and Markov chain Monte Carlo settings
☒	☐	For hierarchical and complex designs, identification of the appropriate level for tests and full reporting of outcomes
☒	☐	Estimates of effect sizes (e.g. Cohen's *d*, Pearson's *r*), indicating how they were calculated

Our web collection on statistics for biologists contains articles on many of the points above.

Software and code

Policy information about availability of computer code

Data collection	Electrophysiological data acquisition - pClamp 9 (Molecular Devices)<br>Confocal image acquisition - confocal laser-scanning microscopes: TCS SP5 and LAS X (Leica) and Zeiss LSM 800 and image acquisition softwares: LAS X (Leica) and Zeiss ZEN.<br>Imaging of gels and blots - ImageQuant LAS 4000 imaging system (GE Healthcare) and capture software ImageQuant LAS 4000 (Fujifilm)<br>NOR studies and three-chamber test recording - EthoVision XT (Noldus Technology)
Data analysis	Electrophysiological data analysis - Clampfit10 (Molecular Devices)<br>Data analysis - Microsoft Excel (Microsoft Windows)<br>Statistical analysis - GraphPad Prism v6 (GraphPad Software, USA) and SigmaPlot v11 (Systat Software, USA)<br>Behavioral analysis - EthoVision XT (Noldus Technology)<br>Image analysis - ImageJ/Fiji (National Institutes of Health, USA) and !marls (OXFORD Instruments).<br>Image deconvolution - Huygens (Scientific Volume Imaging)

For manuscripts utilizing custom algorithms or software that are central to the research but not yet described in published literature, software must be made available to editors and reviewers. We strongly encourage code deposition in a community repository (e.g. GitHub). See the Nature Portfolio guidelines for submitting code & software for further information.

Data

Policy information about availability of data

All manuscripts must include a data availability statement. This statement should provide the following information, where applicable:

- Accession codes, unique identifiers, or web links for publicly available datasets
- A description of any restrictions on data availability
- For clinical datasets or third party data, please ensure that the statement adheres to our policy

All data generated this study are available in the main text and the Supplementary Information file. Source data are provided with this paper.

Research involving human participants, their data, or biological material

Policy information about studies with human participants or human data. See also policy information about sex, gender (identity/presentation), and sexual orientation and race, ethnicity and racism.

Reporting on sex and gender	N/A
Reporting on race, ethnicity, or other socially relevant groupings	N/A
Population characteristics	N/A
Recruitment	N/A
Ethics oversight	N/A

Note that full information on the approval of the study protocol must also be provided in the manuscript.

Field-specific reporting

Please select the one below that is the best fit for your research. If you are not sure, read the appropriate sections before making your selection.

☒ Life sciences ☐ Behavioural & social sciences ☐ Ecological, evolutionary & environmental sciences

For a reference copy of the document with all sections, see nature.com/documents/nr-reporting-summary-flat.pdf

Life sciences study design

All studies must disclose on these points even when the disclosure is negative.

Sample size	No statistical test was employed to predetermine sample size. Our sample sizes were chosen based on previous publications of similar experiments:<br>Cheung, G., Bataveljic, D., Visser, J. et al. Physiological synaptic activity and recognition memory require astroglial glutamine. Nat Commun 13, 753 (2022). https://doi.org/10.1038/s41467-022-28331-7<br>Vasile F, Dossi E, Moulard J, Ezan P, Lecoin L, Cohen-Salmon M, et al. (2022) Pannexin 1 activity in astroglia sets hippocampal neuronal network patterns. PLoS Biol 20(12): e3001891. https://doi.org/10.1371/journal.pbio.3001891<br>Pannasch, U., Freche, D., Dallérac, G. et al. Connexin 30 sets synaptic strength by controlling astroglial synapse invasion. Nat Neurosci 17, 549–558 (2014). https://doi.org/10.1038/nn.3662
Data exclusions	There was no data exclusion
Replication	Replications were carried out successfully. At least 3 animals were used as biological replicates. Independent experiments were performed on individual brain slices, tissue sections or hippocampi obtained from different mice. Details of exact number of replicates carried out for each set of experiments are reported in Figure Legends and Results.
Randomization	Male mice were selected randomly with correct age and genotype. No randomization was acquired. Hippocampal slices were selected randomly after acute slices preparation of tissue sectioning.
Blinding	Blind analysis was performed for behavioral experiments. Blinding was not possible for the other experiments as the experimental conditions were always evident to the experimenters.

Reporting for specific materials, systems and methods

We require information from authors about some types of materials, experimental systems and methods used in many studies. Here, indicate whether each material, system or method listed is relevant to your study. If you are not sure if a list item applies to your research, read the appropriate section before selecting a response.

Materials & experimental systems

n/a	Involved in the study	
	x	Antibodies
	x	Eukaryotic cell lines
x		Palaeontology and archaeology
	x	Animals and other organisms
x		Clinical data
x		Dual use research of concern
x		Plants

Methods

n/a	Involved in the study	
x		ChIP-seq
x		Flow cytometry
x		MRI-based neuroimaging

Antibodies

Antibodies used

Primary antibodies:
Polyclonal rabbit anti-Kir4.1 (IHC 1:100 and WB 1:1000, APC-035, Alomone Labs)
Monoclonal mouse anti-GFAP (1:300, clone G-A-5, G3893, Sigma Aldrich)
Polyclonal chicken anti-GFP (1:500, ab13970, Abcam)
Monoclonal mouse anti-FMRP antibody (15 µg for IP and 1:100 for FISH, 7G1-1, Developmental Studies Hybridoma Bank)
Mouse IgG - Isotype Control (5 µg, ab37355, Abcam)
Mouse anti-beta actin conjugated to HRP (1:2000, ab49900, Abcam)

Secondary antibodies:
Goat anti-mouse IgG conjugated to Alexa 488 (1:1000; A11029, Life Technologies)
Goat anti-rabbit IgG conjugated to Alexa 555 (1:1000, A21429, Life Technologies)
Goat anti-chicken IgG conjugated to Alexa 488 (1:1000, A11039, Life Technologies)
Goat anti-mouse IgG conjugated to Alexa 555 (1:200, A21424, Life Technologies)
Streptavidin Alexa-488 conjugate (1:300, s11223, Invitrogen)
Goat anti-rabbit IgG-HRP (1:2000, CSA2115, Cohesion Biosciences)

Validation

All antibodies used in this study are commercially available and were validated by manufacturer and/or studies cited by the company's website.

Polyclonal rabbit anti-Kir4.1 (IHC 1:100 and WB 1:1000, APC-035, Alomone Labs):
https://www.alomone.com/p/anti-kir4-1/APC-035

Monoclonal mouse anti-GFAP (1:300, clone G-A-5, G3893, Sigma Aldrich)
https://www.sigmaaldrich.com/BE/en/product/sigma/g3893

Polyclonal chicken anti-GFP (1:500, ab13970, Abcam)
https://www.abcam.com/en-be/products/primary-antibodies/gfp-antibody-ab13970

Monoclonal mouse anti-FMRP antibody (15 µg for IP and 1:100 for FISH, 7G1-1, Developmental Studies Hybridoma Bank)
https://dshb.biology.uiowa.edu/7G1-1

Mouse IgG - Isotype Control (5 µg, ab37355, Abcam)
https://www.abcam.com/en-be/products/primary-antibodies/mouse-igg-isotype-control-ab37355

Mouse anti-beta actin conjugated to HRP (1:2000, ab49900, Abcam)
https://www.abcam.com/en-be/products/primary-antibodies/hrp-beta-actin-antibody-ac-15-ab49900

Eukaryotic cell lines

Policy information about cell lines and Sex and Gender in Research

Cell line source(s)	HEK293T cells (human, ATCC CRL-1573)
Authentication	None of the cell lines have been authenticated.
Mycoplasma contamination	Absence of Mycoplasma contamination of the cell line was assessed every 6 months by pcr methods.
Commonly misidentified lines (See ICLAC register)	No commonly misidentified lines were used.

Animals and other research organisms

Policy information about studies involving animals; ARRIVE guidelines recommended for reporting animal research, and Sex and Gender in Research

Laboratory animals	All the experiments were carried out using male mice of<br>wild-type (WT) of C57BL/6j background<br>Fmr1 knockout (KO) mice<br>Fmr1fl/fl: mice containing Cre-excisable loxP sequence in the Fmr1 gene<br>Kir4.1fl/fl: mice containing Cre-excisable loxP sequence in the gene encoding Kir4.1; provided by K. D. McCarthy, University of North Carolina, USA<br>Kir4.1-/-: mice with conditional deletion of Kir4.1 in glia, Kir4.1fl/fl:hGFAP-Cre; provided by K. D. McCarthy, University of North Carolina, USA<br>Both non-transgenic littermate and age-matched WT males were used as control mice.<br>All animals were 21- to 32-day-old males unless otherwise stated. Adult mice were used for behavior experiments at the age of 4-5 months. Mice were housed under 12 h/12 h light/dark cycle, temperature of 22°C, humidity 55% and with food and water available ad libitum.
Wild animals	This study did not involve wild animals.
Reporting on sex	In this study only males were used. The Fmr1 gene is located on the X chromosome and therefore mutations in this gene in males display higher penetrance and severe FXS symptoms in comparison to heterozygous females showing variability in FXS symptoms and milder manifestation. In order to avoid variability due to gender differences we used only male mice and limited the number of used animals following the principles of 3R.
Field-collected samples	This study did not involve samples collected from the field.
Ethics oversight	All procedures on animals strictly followed the guidelines of the European Community Council Directives of January 1st 2013 (2010/63/EU) and French ethic committee (certificate A751901. delivered by the French Ministry of higher education, research and innovation).

Note that full information on the approval of the study protocol must also be provided in the manuscript.

Plants

Seed stocks	N/A
Novel plant genotypes	N/A
Authentication	N/A
